# Big Five personality traits and voting: A systematic review, meta-analysis, and mega-analysis

**DOI:** 10.1177/08902070251383955

**Published:** 2025-10-18

**Authors:** Alexander Georg Stahlmann, Christopher J. Hopwood, Ulrich Orth, Luke David Smillie, Wiebke Bleidorn

**Affiliations:** 1 Department of Psychology, 27217 University of Zurich, Zurich, Swizerland; 2 Department of Psychology, 27210 University of Bern, Bern, Switzerland; 3 School of Psychological Sciences, 2281 The University of Melbourne, Melbourne, Australia

**Keywords:** Big Five, turnout, political, voting, personality

## Abstract

Voting is an important civic behavior that contributes to the vitality of democratic societies. Previous research indicates that personality traits play an important role in voting intentions and behavior, though findings are mixed due to insufficient statistical power and variability in methods and measures. This study synthesizes evidence from a systematic review, leveraging both meta-analysis and mega-analysis to estimate the associations between the Big Five personality traits and voting intentions and behavior. We drew data from 65,036 participants across 17 studies (meta-analysis) and 44,206 participants across 13 studies (mega-analysis). We estimated zero-order correlations between the Big Five traits and voting, and explored moderating effects of personality and voting measures, nationality, gender, and age. We found modest but robust correlations between the Big Five and voting, with effect sizes ranging from .05 to .10. People with stronger intentions to vote were less neurotic and more extraverted, open, agreeable, and conscientious. Low neuroticism was the only trait that predicted voting behavior. Results were somewhat more pronounced in non-U.S. samples. Together, these findings suggest that the Big Five, though modest in effect, are consistent predictors of voting. We discuss implications for future research and applications in fostering voting behavior.

## Plain language summary

This literature review and pooled data analysis shows that broad personality traits such as extraversion or agreeableness are modest but significant predictors of whether people plan to vote. Low neuroticism, or being less prone to worry or stress, stands out as a predictor of actual voting behavior. The present findings help us understand why people differ in their voting behavior and offer avenues to encourage more people to vote, while also preventing efforts to discourage voting based on certain personality traits.

## Personality traits and voting: Findings from a literature review and pooled data analysis

This literature review and pooled data analysis shows that broad personality traits such as extraversion or agreeableness are modest but significant predictors of whether people plan to vote. Low neuroticism, or being less prone to worry or stress, stands out as a predictor of actual voting behavior. The present findings help understand why people differ in their voting behavior and offer avenues to encourage more people to vote, while also preventing efforts to discourage voting based on certain personality traits.

## Personality and voting: A systematic review, meta-analysis, and mega-analysis

Civic engagement is an avenue by which people can contribute to social progress and shape their community’s future (Adler & Goggin, 2005). A prime example is voting, which plays a critical role in the vitality and development of democratic systems, with effectiveness relying heavily on active voting behavior (Flanagan & Levine, 2010; Metzger et al., 2018). However, not everyone votes. Voting rates in Western countries average at about 60% and have shown a concerning decline of approximately 10% over the past six decades (Coppedge et al., 2023). Similarly, people vary in their voting intentions, which have been increasingly marked by ambivalence or negativity, influenced by a range of psychosocial factors, including nationalism, political efficacy, and conspiracy beliefs (Binder & Smith, 1997; Manzetti & Wilson, 2007; Stephanopoulos & McGhee, 2015).

Personality traits, or individual differences in characteristic patterns of thoughts, feelings, and behaviors, have been theorized to play an important role in voting intentions and behavior. The Big Five personality traits (neuroticism, extraversion, openness, agreeableness, and conscientiousness; see John et al., 2008; McCrae et al., 2000) have been associated with life outcomes ranging from educational and occupational achievements to personal relationships and health (Anglim et al., 2020; Barrick & Mount, 1991; Malouff et al., 2010; Poropat, 2009; Strickhouser et al., 2017). Meta-analytic studies have substantiated the associations between the Big Five traits and various aspects of political engagement, including political trust, involvement, and orientation (Bromme et al., 2022; Sibley et al., 2012). Nevertheless, the literature concerning the role of personality traits in voting intentions and behavior remains fragmented and inconclusive, primarily due to methodological variability and limitations in existing research (see Brandt et al., 2022; Furnham & Cheng, 2019; Weinschenk & Dawes, 2018). The goal of this study was to synthesize the existing literature on associations between the Big Five personality traits and voting intentions and behavior.

Understanding these associations is important for two reasons. First, whereas most research on predictors of voting behavior has focused on factors like political views, demographic variables, and national/cultural norms, there has been comparatively less research on the role of personality traits (Blais, 2006; Harder & Krosnick, 2008; Stockemer, 2017). Thus, this study will help situate the predictive value of personality traits within the broader literature on civic engagement and its policy relevance more generally (Bleidorn et al., 2019). Second, knowledge about the predictive effects of psychological factors including personality traits may be helpful for understanding voting patterns and aid in developing strategies to enhance voter participation, while also guarding against efforts to deter specific personality profiles from voting (Burkell & Regan, 2019; Matz et al., 2023).

### Personality and voting

Despite theoretical reasons to expect associations between Big Five traits and voting behaviors, empirical evidence has been inconsistent, with most studies indicating small, non-significant, or mixed effects. Here, we review existing research on the Big Five and voting, while highlighting methodological limitations that could explain mixed results.

#### Neuroticism

Neuroticism describes frequent experiences of negative emotionality, such as anxiety, moodiness, and self-consciousness (Hisler et al., 2020; Widiger & Oltmanns, 2017). Individuals with high levels of neuroticism may exhibit stronger reactions to political stressors and may experience political discourse and the electoral environment as overwhelming or distressing. This heightened emotional sensitivity may lead people to disengage in politics to avoid the perceived emotional discomfort associated with political participation (Gerber et al., 2013; Schoen & Steinbrecher, 2013). Furthermore, individuals with high levels of neuroticism often face personal and professional difficulties, limiting their capacity to engage with political issues due to diminished cognitive and emotional resources (Lahey, 2009; Widiger & Oltmanns, 2017). This is compounded by the tendency to experience more negative life events and instability, factors that may disrupt political participation (Barlow et al., 2014; Magnus et al., 1993).

Conversely, emotional investment in political matters may also spur action, potentially making people more likely to vote as a means of expressing their concerns or alleviating their anger or anxieties (see Brader, 2005; Marcus & MacKuen, 1993). In other words, neuroticism may either have a negative or a positive effect on voting behavior. Indeed, studies have reported a range of effects—from positive to negative. However, most findings tended to be null, indicating no strong or consistent connection between neuroticism and political voting behavior (e.g., Brandt et al., 2022; Furnham & Cheng, 2019; Gerber et al., 2013; Weinschenk & Dawes, 2018). Therefore, the role of neuroticism in political voting remains unclear.

#### Extraversion

Extraversion is characterized by a tendency to seek out social interactions and group activities and to experience positive emotions (Smillie et al., 2019; Wilt & Revelle, 2017). Extraverted individuals are often more involved in community and social activities, which may extend to political engagement (Bromme et al., 2022; Stahlmann et al., 2023). High levels of extraversion may predispose individuals to participate in group-oriented, voting-related activities, such as canvassing or attending political rallies, thus enhancing their likelihood of voting (Omoto et al., 2020). The social nature of these activities aligns well with extraverts’ preference for group interaction, potentially making political participation both a social and civic activity.

Like neuroticism, the empirical evidence for links between extraversion and voting outcomes is mixed. While some studies found a positive correlation, suggesting that extraverts are more likely to engage in voting (e.g., Brandt et al., 2022; Mattila et al., 2011; Weinschenk & Dawes, 2018), other studies reported null findings, indicating no significant correlation between extraversion and voting behavior (e.g., Gerber et al., 2011; Schoen & Steinbrecher, 2013; Wang, 2014; Wang et al., 2017). However, unlike neuroticism, we are aware of no research reporting a negative association between extraversion and voting. Thus, the open question is whether extraversion is a positive or null predictor of voting intentions and behavior.

#### Openness to experience

Openness to experience is characterized by an interest in novel ideas and activities, with individuals showing a preference for intellectual curiosity and exploration (Connelly et al., 2014; DeYoung, 2015). Those who score high in this trait typically show a greater receptiveness to diverse ideas, encompassing (typically left leaning) political ideologies, policies, and practices (Caprara et al., 2006; Sibley et al., 2012). Such intellectual curiosity could be associated with engagement in political issues and participation in elections. Open people may be more likely to gather information about political candidates and policies, fostering a well-informed and active engagement in the electoral process (Mondak et al., 2010; Mondak & Halperin, 2008).

However, similar to extraversion, evidence for an association between openness to experience and voting behavior is inconsistent. Some studies found a positive influence (e.g., Brandt et al., 2022; Mattila et al., 2011; Weinschenk & Dawes, 2018), but a larger number reported no significant correlation (e.g., Gerber et al., 2011; Schoen & Steinbrecher, 2013; Wang, 2014; Wang et al., 2017).

#### Agreeableness

Agreeableness involves a consideration for others’ feelings and needs, often manifested in communal goals and prosocial behaviors (Graziano & Eisenberg, 1997; Habashi et al., 2016). Individuals high in agreeableness might view voting as a moral responsibility and an extension of their commitment to communal well-being. Their inherent focus on others’ needs and community welfare could translate into a higher propensity for civic engagement, including participating in elections (Graziano & Eisenberg, 1997; Graziano & Tobin, 2009).

However, similar to the patterns seen with other traits, research in this area has yielded mixed outcomes, with some studies reporting positive or negative effects, but most revealing no significant association (e.g., Brandt et al., 2022; Furnham & Cheng, 2019; Gerber et al., 2013; Weinschenk & Dawes, 2018). Therefore, the role of agreeableness in voting remains unclear.

#### Conscientiousness

Finally, conscientiousness refers to an individual’s sense of duty, precision, need for order, and diligence (Roberts et al., 2014). Those high in conscientiousness are typically characterized by adherence to both social and personal norms, including those pertaining to civic responsibilities. This sense of duty and commitment to norms may be associated with a greater inclination to vote, which might be viewed as a civic obligation (Gerber et al., 2011; Mondak & Halperin, 2008).

But empirical findings have again been inconsistent. Whereas some research has found a positive correlation (Furnham & Cheng, 2019; Weinschenk & Dawes, 2018), others have actually found the opposite, that individuals with lower levels of conscientiousness voted more frequently (Gerber et al., 2011, 2013). Still other studies reported no significant link between conscientiousness and voting behavior (e.g., Schoen & Steinbrecher, 2013; Sindermann et al., 2021; Wang et al., 2017). Thus, the role of conscientiousness in voting behavior remains unclear.

### Potential reasons for mixed evidence on personality traits and voting

Overall, the existing evidence paints a very unclear picture of the role the Big Five personality traits may play in voting intentions and behavior. It is possible that there are robust effects that are masked by methodological problems, such as low statistical power or measurement-related issues. Mixed findings may also indicate the presence of moderators such as the distinction between voting intentions and actual voting behaviors, and the sociodemographic characteristics of the study participants, including variables like age, gender, and cultural background. We review each of these potential factors presently.

#### Small effects and power

Associations between personality traits and behavioral attitudes, intentions, or actual outcomes are often described as “small” and thus hard to detect (*r* ∼.20; see Gignac & Szodorai, 2016; Hemphill, 2003; Noftle & Robins, 2007). The detection issue is further compounded in the study of the link between personality and voting, where existing evidence points to even smaller effect sizes (*r* ∼.05 and .10). Moreover, the measures used in these studies are mostly brief and of relatively low reliability. Small effects are unlikely to be detected with relatively unreliable measures without very large samples. Given the significant variation in sample sizes across studies on associations between personality traits and voting, it is plausible that some null findings and part of the mixed body of evidence can be attributed to insufficient power.

It is important to note that statistically small effects can nevertheless be practically important (Götz et al., 2022; Ozer & Benet-Martínez, 2006). For instance, the effect of aspirin on reduced risk of cardiovascular disease is very small (Odds Ratio ∼1.1; Funder & Ozer, 2019). However, this means if 10,000 individuals at risk take aspirin, 85 fewer will have heart attacks. Unlike heart attacks, voting affects all people living in a democratic society. If a personality trait like extraversion influenced voting as aspirin does heart health, targeting voters high in this trait could yield more benefits than targeting those with average or below-average levels. For instance, a political party launching a strategic campaign to sway voters, targeting 20 million eligible voters with a 5% success rate, could secure approximately 610,000 additional votes by random sampling.^1^ However, focusing on individuals with higher extraversion could improve the success of the campaign. An effect size comparable to that of aspirin would result in 632,400 additional votes rather than 610,000.^2^ Conversely, targeting a group low in extraversion might only yield 587,700 additional votes, marking a significant difference of 44,700 votes in favor of effective personality targeting.^3^ This difference could be decisive in tightly contested elections where victory hinges on slim vote margins. In this study, we will use integrative data analysis to generate the sufficient statistical power needed to discern these potentially modest but significant effects.

#### Personality assessment length

Researchers have used a variety of assessment measures to study how personality is related to voting. These range from very brief instruments like the Ten Item Personality Inventory (TIPI; Gosling et al., 2003) to medium-length ones such as the 44-item Big Five Inventory (BFI; John et al., 1991), or long-form assessments such as a 180-item version of the NEO Personality Inventory (e.g., Costa & McCrae, 1992; Mattila et al., 2011; see). Longer instruments are generally more reliable (Henson, 2001; Revelle & Condon, 2019) and thus studies using longer instruments tend to provide more precise estimates, have smaller standard errors, and narrower confidence intervals (Kretzschmar & Gignac, 2019; Nunnally & Bernstein, 1994). Hence, studies that used longer measures may be more likely to detect the anticipated small effects.

Here, we will explore the number of items per personality trait as a continuous moderator of the personality-voting link, with reference levels set at 2 items and 9 items per Big Five domain trait. These benchmarks align with the item counts in the most frequently used brief personality inventories: TIPI (2 items per trait) and BFI (approximately 9 items per trait). Notably, the length of the instrument does not necessarily affect the predictive validity of a scale, and we did not expect to find larger effects with longer measures (Heene et al., 2014; Thalmayer et al., 2011). Yet, our anticipation was that these more comprehensive tools might lead to a reduction in standard errors, consequently enhancing the likelihood to precisely detect small effects.

#### Voting attitudes, intentions, and behaviors

Whereas some studies have assessed actual *voting behavior* (“I have voted during the last presidential election”), others have assessed *voting intentions* (“I will vote at the next presidential election”) or *voting attitudes* (“I believe voting is important”). Intentions and attitudes are similar to personality traits in that they are more abstract than actual behavior, and this may increase correlations between measures of personality and voting. However, attitudes and intentions do not always materialize into actions (Sheeran & Webb, 2016; Taylor & Todd, 1995). Thus, we aimed to examine whether associations between personality and voting depend on whether voting was measured as a behavior, attitude, or intention. We hypothesized that attitudes exhibited the strongest link, followed by intentions, and then behaviors across different personality traits. Intention and attitude measures are not only conceptually and methodologically more similar to personality trait measures, they have been also theorized to mediate the links between broad traits and behavior (Bleidorn, Stahlmann, & Hopwood, 2025; Hopwood et al., 2022).

#### Dichotomous and continuous measures of voting

Another difference in the way voting has been measured across studies has to do with measurement scaling. Some studies employed continuous scales, while others used dichotomous scales, which could have potentially attenuated statistical power (DeCoster et al., 2009; Fedorov et al., 2009; MacCallum et al., 2002). We compared Big Five-voting associations across studies that have used these two kinds of scales and expected that continuous scales measuring voting intentions and behaviors would result in larger correlations compared to dichotomous scales.

#### Sociodemographic variables

Finally, sociodemographic and cultural differences might account for some of the variance in effect sizes. For instance, a recent study by Huber et al. (2021) showed that, even though conscientiousness did not predict voting in the United States, it was a significant predictor in the United Kingdom. Another potential moderator may be a person’s life stage. Lifespan theories of aging suggest that certain developmental tasks, such as voting, may be perceived as more relevant when individuals enter middle age and begin to feel a responsibility for the next generation (Erikson, 1959; Freund & Baltes, 2002; McAdams, 2001). Consequently, individuals may be most likely to express their personality through political activities, including voting, during middle and older adulthood, which may lead to higher correlations than in other life stages. Furthermore, gender has been identified as a significant influence on voting turnout in previous studies and has sometimes emerged as a moderator of the link between personality traits and voting behavior, highlighting the need to explore potential gender-specific patterns in how personality traits influence political engagement (Stahlmann et al., 2023; Wang, 2014; Weinschenk et al., 2018).

In the present study, we examined age as a continuous moderator, and gender (male/female) and nationality (U.S./other) as categorical moderators of the Big Five-voting link. To ease interpretation across age groups, we selected 25 years, 50 years, and 75 years as our reference levels. These benchmarks correspond to established life stage classifications commonly used in studies within the intersection of personality and lifespan psychology (e.g., Bleidorn & Hopwood, 2024; Costa & McCrae, 1976; Freund & Blanchard-Fields, 2014): younger adults (18–34 years), middle-aged adults (35–64 years), and older adults (65–90 years). While we anticipate the most pronounced effects among middle-aged adults, we did not have hypotheses about the effects of gender and nationality.

### Synthesizing existing results

To estimate the small effects of Big Five personality traits on voting intentions and behaviors and to assess methodological influences, robust and integrative data analysis methods are essential. Our approach encompasses two key methodologies: meta-analysis and mega-analysis.

Meta-analysis, a well-established technique, aggregates effect sizes from various studies at the sample level. Its strengths lie in the ability to combine findings from a broad spectrum of studies without requiring raw data, as relevant information is typically accessible through published articles and supplementary materials. However, meta-analysis is susceptible to biases like selective reporting and issues with handling outliers or rounding errors, which can be particularly challenging when dealing with small effect sizes (Borenstein et al., 2021; Cafri et al., 2010; Hohn et al., 2019). Thus, while meta-analysis provides a valuable perspective on the personality-voting connection, it may yield somewhat limited results because the quality of the studies may vary and because of potential publication biases.

Mega-analysis, on the other hand, involves pooling individual-level data, standardizing measures across studies, and applying uniform analytical techniques to the raw data from existing studies. This approach offers increased statistical power, facilitating a more accurate effect size estimation and enhanced capability for moderation testing (see, e.g., Beck & Jackson, 2022). Critically, the rigorous use of mega-analytic techniques requires sufficient raw data and the standardization of different measures across samples, which can introduce variability and complicate the interpretation of mega-analytic results (Burke et al., 2017; Curran & Hussong, 2009). Consequently, while mega-analysis addresses some limitations of meta-analysis by offering greater power, it also presents its own challenges, such as the exclusion of studies where data access is unfeasible, or when measures are incompatible with the research questions.

Given that both meta- and mega-analytic approaches have strengths and limitations, a combined approach should prove particularly effective in addressing their individual limitations, especially when dealing with small effect sizes and when complete access to all individual studies is not feasible. Here, we adopted a dual-study approach, applying both meta-analysis and mega-analysis to a dataset gathered through systematic review. The integration of these two approaches aims to provide a richer understanding of how the Big Five correlate with voting intentions and behavior, including the exploration and testing of hypotheses about potential moderators. The intention of this integrated analysis is to generate robust estimates, enhancing their reliability and the potential for replication in subsequent individual studies.

### The present study

In this study, we used data from a systematic review to investigate the connections between the Big Five personality traits and voting. Our research methodology and analyses involved three distinct phases: (1) a systematic review process, (2) meta-analyses of the links between the Big Five and voting, and (3) mega-analyses of the links between the Big Five and voting. We consider the combination of meta- and mega-analyses as analogous to a sensitivity analysis (Nosek et al., 2022; Simonsohn et al., 2020). Our primary goal was to identify robust patterns of results that hold true across both analytical approaches, thus providing the most comprehensive and replicable estimates of the Big Five-voting links.

Our literature review suggested that results were consistently positive for openness and extraversion when significant. Conscientiousness and agreeableness showed similar trends, albeit with sporadic negative correlations. The evidence was more mixed for neuroticism, with a 2:1 ratio of negative to positive correlations. Using a benchmark of *r* ∼.05 and .10 for *very small* to *small* effects, respectively (see Bosco et al., 2015; Gignac & Szodorai, 2016), we thus preregistered the following hypotheses (see https://osf.io/4z6s9):

• H1a–H1e: The correlations between Big Five personality traits and voting will be small (*r* ≥ .05). The correlations with (a) openness, (b) conscientiousness, (c) extraversion, and (d) agreeableness will be positive and the correlation with (e) neuroticism will be negative.

Beyond estimating zero-order correlations, we also examined the moderating effects of various factors. These included the type of assessment tool (brief versus longer), the conceptualization and operationalization of voting (intention versus behavior, dichotomous versus continuous scaling), and sociodemographic variables (nationality, gender, and age). We additionally preregistered the following hypotheses:

• H2a–H2e: Personality-voting correlations will be strongest for voting attitudes and continuous scales, less strong for voting intent, and weakest for reported voting and dichotomous scales.
• H3a–H3e: Personality-voting correlations will be strongest for middle-aged adults (35–64 years) compared to other age groups.

In further considering the assessment tool used (brief/longer) as a potential moderator, we did not anticipate finding larger effects with longer measures. However, we anticipated that these more comprehensive tools might reduce standard errors, thereby increasing statistical power. Regarding the potential moderating roles of nationality and gender, we did not formulate specific predictions. Instead, we planned to explore their potential influence on the personality-voting link.

After estimating all meta-analytic and mega-analytic models, we consolidated the evidence to test hypotheses. If both types of analysis concurred on a significant effect, such as a small positive correlation (*r* ≥ .05) between openness and voting, we deemed our hypothesis confirmed. If only one type of analysis suggested such an effect, we viewed our hypothesis as partially supported. If both analyses indicated no effect or contradictory effect sizes, we regarded our hypothesis as unsupported.

### Transparency and openness

We report how we determined our sample size, all data exclusions (if any), all manipulations, and all measures in the study.

We adhered to the PRISMA 2020 guidelines for systematic reviews (Page et al., 2021). All data, analysis code, and research materials (including our coding scheme) are available on OSF (https://osf.io/mq2n5). The review project was also preregistered on OSF, with an initial version (https://osf.io/3abwz) and a subsequent revised version (https://osf.io/u9d68).

We adhered to the MARS guidelines for meta-analytic reporting (Appelbaum et al., 2018). All meta-analytic data, analysis code, and research materials (including our coding scheme) are available on OSF (https://osf.io/mq2n5). The mega-analytic harmonization and analysis code are available in the same OSF project. The mega-analytic dataset cannot be shared, as it was compiled from multiple databases and repositories across different countries, each subject to its own legal regulations. We obtained permission to use these data exclusively for the purposes of this study and formalized this agreement through the required documentation. Nonetheless, access to the original datasets should be attainable for other researchers interested in conducting independent analyses. Data were analyzed using R, version 4.3.0 (R Core Team, 2023) and the packages *metafor*, version 4.4-0 (Viechtbauer, 2023), *clubSandwich*, version 0.5.10 (Pustejovsky, 2023), and *lme4*, version 1.1-35.1 (Bates et al., 2023). The meta-analysis and mega-analysis were also preregistered on OSF (https://osf.io/4z6s9). The preregistration was submitted after conducting the systematic review, effect size coding, and data retrieval but before undertaking the meta-analysis and mega-analysis.

## Systematic review

**Figure 1.**
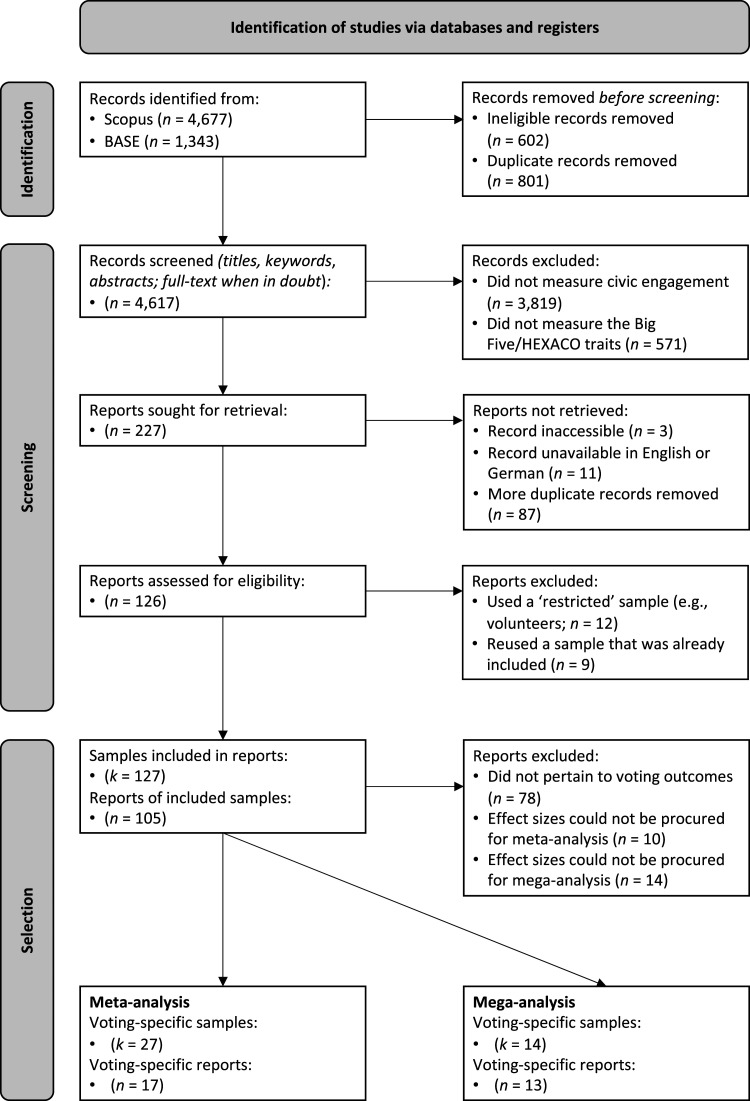
PRISMA flow diagram, summarizing the literature search process and the selection of eligible studies.

The data used in the meta-analysis and mega-analysis were derived from a systematic review that compiled studies examining the links between Big Five or HEXACO personality traits and various indicators of civic engagement, including voting. Here, we detail the comprehensive search process for all indicators of civic engagement. This is because the segregation of data specific to voting occurred only after completing all essential steps—identification of studies, screening, eligibility, inclusion of studies, and coding of studies. A complete PRISMA flow diagram, summarizing the entire literature search process and the selection of eligible studies, is available in Figure 1.

Given the bibliographic nature of the study, ethical review and approval were deemed unnecessary in accordance with local legislation and institutional guidelines.

### Identification of studies

The systematic review targeted empirical, quantitative studies published in either English or German—the working languages of the authors. The studies were sourced through a search of article titles, abstracts, and indexed keywords in Scopus (primary database), along with the titles and keywords indexed in BASE (grey literature).

The following search terms were used: (*personality OR* “*Big Five*” *OR* “*five factor model*” OR HEXACO OR extraver* OR introver* OR surgency OR agreeable* OR conscientious* OR openness OR “*open to experience*” *OR* “*open to experiences*” OR intellect OR neurotic* OR “*emotional stability*” *OR* “*emotionally stable*” *OR emotionality OR honesty OR humility*) *AND* (volunteeri* OR charit* OR philanthrop* OR donat* OR “don* blood” *OR* “blood don*” *OR* “don* organ*” *OR* “organ don*” *OR* “*vot*” *OR* “politic* particip*” *OR* “politic* behav*” *OR* “politic* involve*” *OR* “politic* engage*” OR vaccin*).^4^ Two search queries were executed, the first on February 25, 2022, and the second on March 1, 2023. The second query was necessary after recognizing the omission of certain relevant cited articles due to the initial search terms’ lack of comprehensiveness. Specifically, the following terms pertaining to voting were deemed missing from the first query: “politic* particip*,” “politic* behav*,” “politic* involve*,” and “politic* engage*.”

These specific search terms were chosen because they were commonly used in key prior studies on personality and voting (e.g., Mondak et al., 2010; Mondak & Halperin, 2008). Some terms (e.g., “politic* engage*”) were not central to our core constructs but were included to avoid missing studies in which voting intentions or behaviors were only secondary outcomes or control variables. We tested the final set of keywords repeatedly to ensure that it captured all studies already known to the author team.

The search results were acquired automatically from Scopus through Christopher Belter’s Scopus Search API (https://github.com/christopherBelter/scopusAPI), while BASE search results were gathered manually. The R script that was used for the study retrieval is available on OSF (see https://osf.io/x9pzh). A total of 4,677 studies were identified on Scopus and 1,343 on BASE. Of these, 1,403 were automatically excluded as they were flagged as conference reviews, editorials, letters, notes, reviews, musical notations, maps, audios, images/videos, software, datasets, or because they were duplicates of already included studies.

### Screening, eligibility, and inclusion of studies

Each of the remaining 4,617 studies was independently screened by the first author or one of four graduate students. Their focus at this stage was to ascertain whether the studies included measures of Big Five or HEXACO personality traits and civic engagement, with a preference for inclusion when in doubt. Both self-reported and peer-reported data were eligible for inclusion, and civic engagement indicators could be measured using dichotomous or continuous scales, as long as they corresponded to behavioral attitudes, intentions, or actual behaviors. The study design could also vary, provided it was either correlational (cross-sectional, longitudinal, and panel studies) or experimental (lab and field studies). Meta-analyses, clinical trials, and single-case studies were excluded, as well as studies reporting on economic games, such as the Dictator Game or Ultimatum Game, to prevent redundancy with another recent meta-analysis (Thielmann et al., 2020).

The screening was primarily based on the titles, keywords, and abstracts, turning to the full text only when uncertainties arose. During this process, the raters remained blind to publication metrics provided by Scopus and BASE, which included journal titles, study authors, and citation counts. To ensure high interrater consistency, a calibration process was undertaken prior to the screening phase. This process involved the repeated assessment of 100 study batches until the raters achieved an agreement rate exceeding κ = 95%. This calibration process was carried out three times, resulting in the collective appraisal of 300 studies. In the end, an additional 4,390 studies were excluded due to the absence of measures pertaining to both Big Five or HEXACO personality traits and civic engagement, or because they failed the other eligibility criteria.

Of the remaining 227 studies, three were excluded because they were unattainable despite best efforts, 11 were excluded because they were not available in English or German, and 87 were excluded because they were duplicates of previously included studies. The first author and two of the graduate students who had been involved in the previous screening stage independently assessed the full texts of the remaining 126 studies, ensuring that each study was reviewed by all three raters. During this process, an additional 21 studies were excluded as they were based on populations that were expected to have high scores in one or all civic engagement indicators (for example, samples only comprising individuals who had voted in a previous election) or because they reused a sample already included in a different study. The final selection comprised 105 studies, with 27 of these including measures of voting. Table 1 gives an overview of these studies, including demographic information, the conceptualization/operationalization of voting, and the reported direction of effects, if any were detected. The complete data sheet detailing study codes, variables, and effect sizes is also available on OSF (see https://osf.io/zk6m8).

**Table 1.** Summary of studies identified in the systematic review, categorized by inclusion in meta-analysis, mega-analysis, or both.

No.	Code	Study	Country	*N*	Women	Age *M*	Concept.	Operat.	*N*	E	O	A	C	Use	Data	Sampling
1	854	Schoen and Steinbrecher (2013)	DEU	2,115	52.8%	50.2	Behavior	Dichotomous	-	0	0	+	0	Both	GLES 2009	Random
2	877	Gerber et al. (2013)	USA	1,444	55.2%	31.0	Intention	Continuous	0	0	0	0	0	Both	Study sample S1	Convenience
2	877	Gerber et al. (2013)	USA	3,382	58.9%	60.5	Behavior	Dichotomous	-	0	0	-	-	Both	Study sample S2	Convenience
3	928	Wang (2014)	USA	2,606	57.8%	53.1	Intention	Continuous	-	0	0	0	0	Both	ANES 2010	Representative
4	1660	Furnham and Cheng (2019)	GBR	9,790	50.7%	50.0	Behavior	Dichotomous	+	+	+	+	+	Both	NCDS	Population
5	2217	Sindermann and Montag (2023)	DEU	3,325	60.3%	37.7	Intention	Dichotomous						Both	Study sample S3	Convenience
6	2453	Sindermann et al. (2021)	DEU	4,286	53.8%	38.0	Intention	Dichotomous	0	0	+	+	0	Both	Study sample S4	Convenience
7	2524	Brandt et al. (2022)	DEU	2,687	61.6%	39.8	Intention	Dichotomous	-	+	+	+	0	Both	BIJU: Wave 8	Random
8	2750	Gerber et al. (2011)	USA	3,367	51.3%	50.2	Intention	Dichotomous	0	0	0	0	0	Both	CCAP 2008	Weighted
8	2750	Gerber et al. (2011)	USA	3,367	51.3%	50.2	Intention	Dichotomous	0	0	0	0	0	Both	CCAP 2008	Weighted
8	2750	Gerber et al. (2011)	USA	3,367	51.3%	50.2	Behavior	Continuous	-	0	0	-	-	Both	CCAP 2008	Weighted
8	2750	Gerber et al. (2011)	USA	3,367	51.3%	50.2	Behavior	Continuous	0	0	0	0	0	Both	CCAP 2008	Weighted
9	3038	Smith et al. (2019)	USA	800	51.2%	47.6	Behavior	Continuous						Both	Study sample S5	Representative
10	758	Gallego and Oberski (2012)	ESP	3,459	52.2%	48.6	Behavior	Dichotomous						Mega	PEALE 2009	Weighted
11	2785	Verhulst (2012)	USA	1,349	61.2%	57.2	Intention	Continuous						Mega	MTPS	Convenience
12	2843	Weinschenk (2013)	USA	1,500	52.4%	48.4	Intention	Dichotomous						Mega	AB 2010	Weighted
13	3024	Weinschenk et al. (2019)	DEU	4,096	58.4%	23.0	Behavior	Dichotomous						Mega	TwinLife Wave 1	Random
14	579	Anderson (2009)	USA	818			Behavior	Dichotomous	-	0	0	0	0	Meta		
15	678	Mattila et al. (2011)	FIN	212		36.0	Behavior	Continuous	0	+	0	+	0	Meta		
15	678	Mattila et al. (2011)	FIN	212		36.0	Behavior	Continuous	0	+	0	+	+	Meta		
16	1421	Wang et al. (2017)	KOR	1,245	54.0%	50.5	Behavior	Continuous	0	0	0	0	0	Meta		
17	1912	Wang et al. (2019)	TWN	839	50.0%	44.2	Behavior	Dichotomous	0	0	0	0	0	Meta		
18	2707	Mondak and Halperin (2008)	USA	404			Behavior	Dichotomous	0	0	0	0	0	Meta		
18	2707	Mondak and Halperin (2008)	USA	1,338			Behavior	Dichotomous	0	0	0	0	0	Meta		
19	2732	Mondak et al. (2010)	USA	1,062			Behavior	Dichotomous	+	0	+	0	0	Meta		
20	2788	Cooper et al. (2013)	USA	760	68.0%	47.6	Behavior	Dichotomous	0	0	0	+	+	Meta		
21	3115	Huber et al. (2021)	BRA	1,086			Behavior	Continuous						Meta		
21	3115	Huber et al. (2021)	KOR	944			Behavior	Continuous						Meta		
21	3115	Huber et al. (2021)	RUS	1,145			Behavior	Continuous						Meta		
21	3115	Huber et al. (2021)	GBR	1,064			Behavior	Continuous						Meta		
21	3115	Huber et al. (2021)	USA	1,161			Behavior	Continuous						Meta		
22	903	Turska-Kawa (2013)	POL	726	52.6%		Behavior	Continuous						None		
23	1385	Weinschenk and Panagopoulos (2014)	USA	724	46.0%	34.0	Intention	Continuous	0	0	0	0	0	None		
24	1467	Weinschenk and Dawes (2018)	USA	724	46.0%	34.0	Attitude	Continuous	-	+	+		+	None		
25	1690	Pruysers et al. (2019)	CAN	371	58.0%	49.2	Attitude	Continuous						None		
26	1880	Ivanchenko et al. (2019)	UKR	1,247	59.0%	25.8	Behavior	Continuous	+	0	0	0	+	None		
27	2504	Lynch (2008)	USA	63	70.0%	22.0	Behavior	Dichotomous						None		
27	2504	Lynch (2008)	USA	63	70.0%	22.0	Intention	Continuous						None		

*Note.* “Code” refers to the study code assigned in the systematic review (https://osf.io/zk6m8). “Country” uses ISO 3166-1 alpha-3 codes. *N* = Number of individuals, Women = Percentage of women, Age *M* = Average age. These demographic details were computed for studies included in the mega-analysis or both meta- and mega-analysis. For studies used solely for meta-analysis, information was retrieved from the literature. “Concept.” Is an abbreviation for voting conceptualization. “Operat.” Is an abbreviation for voting operationalization. “NEOAC” indicates the reported effects for Big Five personality traits, if any were identified: *N* stands for neuroticism (or negative emotionality versus emotional stability), E for extraversion (or surgency), O for openness to experience (or culture/intellect), C for conscientiousness, and A for agreeableness, and. Symbols (+), (−), and (0) represent evidence of a positive link, a negative link, and no link, respectively. Empty cells imply missing information. Study samples from S1 to S5 primarily consist of convenience samples, gathered specifically for these studies, in contrast to the samples used in the other studies, which were panel data.

## Meta-analysis

After completing the systematic review, we identified a subset of studies with sufficient data for inclusion in the meta-analysis. Following the coding of these studies, we conducted the meta-analysis and present the outcomes of this initial phase in the following sections.

### Coding of studies

From the 27 studies identified in the systematic review, the first author and the two graduate students used a standardized coding manual (see https://osf.io/4hrz7) to code the (1) sample size, (2) effect size, (3) the type of effect size statistic, (4) the number of items used for the Big Five measurement, (5) the conceptualization of voting (attitude/intention/behavior), (6) the operationalization of voting (dichotomous/continuous), (7) the geographic region where the sample was collected, (8) the percentage of female participants, and (9) the average age of participants. In the single longitudinal study identified, we only included the initial measurements of personality traits measured at age 33 and voter turnout at age 36, while excluding subsequent personality assessments at ages 42 and 50 (Mattila et al., 2011). All coded variables and statistics were then cross-checked amongst the coders for consistency. Discrepancies in coding were resolved through discussion until a consensus was reached.

Raw effect sizes suitable for meta-analysis, that is, Pearson’s correlations, standardized mean differences, or odds ratios, were not reported in 24 studies (88.89%). In such instances, attempts were made to contact the respective authors or calculate the missing effect sizes from the data and supplementary materials available. The R script that includes these calculations is available on OSF (see https://osf.io/x9pzh). Effect sizes were obtainable from the authors in nine instances and could be computed from the available data in six cases. However, effect sizes could not be procured for the remaining nine studies, leaving 18 studies as the provisional basis for the meta-analysis. Upon further analysis of the moderator variables, we noticed that most studies employed measures of voting intention or behavior, whereas only one study used an attitude measure of voting (Weinschenk & Dawes, 2018). To maintain consistency in voting operationalization and avoid confusion, we decided to exclude this study. Consequently, the meta-analysis was based on a final sample of 17 studies that included measures of voting intention or behavior.

### Summary of the literature

Table 2 summarizes key statistics for the 17 studies included in the meta-analysis. These studies encompass 27 datasets, owing to some studies reporting different Big Five measures or multiple voting measures (e.g., Gerber et al., 2011; Gerber et al., 2013; Mattila et al., 2011).

**Table 2.** Descriptive statistics of data included in the meta-analysis, mega-analysis, or both.

	Meta-analysis	Mega-analysis	Included in both types of analyses	
			Systematic review data	Mega-analytic data
Total sample size (*N)*	65,036	44,206	42,598	33,802
Total study size (*k)*	17	13	9	9
Individual dataset size (*n)*				
Mean (*M)*	1,871	3,158	2,811	3,380
Standard deviation (*SD*)	1,716	2,192	1,915	2,472
Minimum (*Min*)	212	800	800	800
Maximum (*Max*)	7,135	9,790	7,135	9,790
Percentage of women (*%*)				
Mean (*M)*	56.0	54.8	54.6	54.4
Standard deviation (*SD*)	6.0	4.0	4.7	3.9
Minimum (*Min*)	50.0	50.7	50.0	50.7
Maximum (*Max*)	68.0	61.6	62.0	61.6
Average age (years)				
Mean (*M)*	42.8	46.2	42.5	46.8
Standard deviation (*SD*)	9.3	9.5	10.5	7.9
Minimum (*Min*)	22.8	23.0	22.8	31.0
Maximum (*Max*)	52.4	60.5	52.4	60.5
Number of items				
Mean (*M)*	6.4	4.4	4.9	4.6
Standard deviation (*SD*)	6.8	3.3	3.6	3.4
Minimum (*Min*)	1.0	1.0	1.0	1.0
Maximum (*Max*)	36.0	10.0	10.0	10.0
Voting conceptualization as intention (*%*)	22.2	46.7	38.5	54.5
Voting operationalization as dichotomous scale (*%*)	48.1	66.7	46.2	63.6
Geographic locations in the U.S. (*%*)	51.9	50.0	61.5	50.0

*Note.* The descriptive statistics for data included in both meta- and mega-analyses vary depending on the source. When derived from the systematic review, they reflect information presented in the included studies. Conversely, when based on our computations, they stem from the data available for meta-analysis. The final three rows indicate the percentages of studies conceptualizing voting as intention (versus behavior), operationalizing voting on a dichotomous scale (versus a ratio scale), and focusing on geographic locations in the U.S. (versus other countries).

The average sample size across individual studies was 1,871 participants (*SD* = 1,716, *Range* = 212–7,135). Gender distribution was approximately balanced in most studies, with 56.0% women on average and a maximum of 68.0% in Cooper et al. (2013). Participants were mostly middle-aged adults, with an average age of 42.8 years, and a minimum age of 22.8 years in one study (Brandt et al., 2022). Most studies used scales with one or two items per trait (i.e., the FIPI or TIPI; Gosling et al., 2003) or the BFI (eight to ten items per trait; John et al., 1991) to measure the Big Five personality traits. An exception was the study by Mattila et al. (2011), which employed a 36-item-per-trait version of the NEO inventory. There was a slight preference for measuring voting behavior over voting intentions in the included studies. However, the distributions of voting operationalization (dichotomous/continuous) and geographic location (U.S./other) were relatively balanced. Outside the United States, datasets were sourced from various countries, including Germany (Brandt et al., 2022; Schoen & Steinbrecher, 2013; Sindermann et al., 2021; Sindermann & Montag, 2023), Great Britain (Furnham & Cheng, 2019; Huber et al., 2021), Finland (Mattila et al., 2011), South Korea (Huber et al., 2021; Wang et al., 2017), the Island of Taiwan (Wang et al., 2019), Brazil (Huber et al., 2021), and Russia (Huber et al., 2021).

### Analysis plan

Consistent with broader research trends, the systematic review indicated that the literature about the links between the Big Five and voting can be best understood through a three-level model (see Cheung, 2014; Pastor & Lazowski, 2018). In this model, individual data are nested in measures of different personality traits or voting conceptualizations/operationalizations, which are then nested within individual studies.^5^ We aimed to mirror this nested structure to separate and quantify variations both within individual studies and across the larger research corpus. In the meta-analytic framework, we hence implemented and tested three-level models (Assink & Wibbelink, 2016; Cheung, 2014).

First, we transformed all raw effect sizes into Fisher’s z-scores and estimated their standard errors (Borenstein et al., 2021; Fisher, 1921). Then, as a first sensitivity analysis, we fitted five intercept-only three-level Correlated and Hierarchical Effects (CHE) models with robust variance estimation to the data, setting different constants for within-study correlations (*ρ* = .00, .20, .40, .60, .80). This approach was chosen due to the presence of multiple effect sizes per study and the cultural diversity of the studies, making CHE models more suitable than random-effects models (Assink & Wibbelink, 2016; Cheung, 2014). The analysis showed that varying the constant had minimal impact on the model estimates, with differences emerging only at the third decimal place. However, given the likelihood of some correlation between effect sizes within the same study, and considering that measurement error can produce small correlations even when the true correlation is zero, we opted for the *ρ* = .20 model, which assumes within-study correlations of the Big Five traits to settle around .20, due to common-method effects (Borenstein et al., 2021; Schmidt & Hunter, 2014).

Subsequently, we fitted a series of three-level CHE models with robust variance estimation to the data, using restricted maximum-likelihood estimation (REML) alongside Hartung-Knapp-Sidik-Jonkman adjustment to account for potential overestimation of effect size precision due to variability among studies (Borenstein et al., 2021; Knapp & Hartung, 2003; Sidik & Jonkman, 2002). Additionally, we corrected the confidence and prediction intervals by employing a sandwich estimator for standard errors and the Satterthwaite approximation for critical values (Satterthwaite, 1946; Tipton & Pustejovsky, 2015). The initial model was structured to treat the Big Five personality traits as categorical moderators influencing voting outcomes, with each trait represented by a separate dummy variable (0 or 1) to specify the corresponding effect size in a long-format dataset. We then progressively included additional moderators in the analysis: (1) the number of items used for personality measurement (brief/longer), (2) the conceptualization and operationalization of voting (intention/behavior, and dichotomous versus continuous scaling), and (3) sociodemographic variables (nationality, gender, age). As a final step, we converted the model statistics back into correlations and reported point estimates, along with corrected 95% confidence intervals and further model diagnostics, such as *I*^2^ (Borenstein et al., 2021; Fisher, 1921).

### Robustness checks and deviations from preregistration

All of our analyses were preregistered and grounded in a limited set of a priori hypotheses, and thus we did not apply formal multiple-comparison corrections (e.g., Bonferroni adjustments). In our baseline models, we tested five primary trait–outcome associations—one for each Big Five personality trait—each specified in advance. Although our moderator models involve additional parameters (including interaction effects), these analyses were also determined a priori, thereby minimizing the risk of spurious findings that commonly occur in exploratory examinations (Perneger, 1998; Rothman, 1990). In this context, overly conservative adjustments would unduly elevate the risk of Type II errors (i.e., false negatives), potentially obscuring genuine theoretical effects (Gelman et al., 2012; McShane et al., 2019). Consequently, we emphasize effect size estimation by reporting point estimates along with robust 95% confidence intervals, and we employ robust variance estimation to account for dependencies among effect sizes (Tipton & Pustejovsky, 2015). This approach is consistent with meta-analytic best practices, which favor estimation over reliance on dichotomous significance thresholds (Borenstein et al., 2021; Schmidt & Hunter, 2014), thereby ensuring that our inferences reflect both the magnitude and precision of the observed effects.

To assess the possible influence of outliers, we visually inspected forest plots and calculated Cook’s distance and DFFITS for each Big Five trait. Due to the lack of established cutoffs for influential outliers, we performed sensitivity analyses with and without studies showing extreme deviations or high Cook’s distance and DFFITS values (Viechtbauer & Cheung, 2010). We retained these studies in our models when sensitivity analyses yielded similar results (these ancillary analyses are available on OSF; https://osf.io/xjuqm). When significant differences, like effect direction changes or new statistical significance, were observed, we included both sets of analyses in our results. For small-study effects, we examined contour-enhanced funnel plots and Egger’s regression test for each trait (Egger et al., 1997; Peters et al., 2008). In cases of asymmetry or indicated small-study effects, we applied the PET-PEESE method for bias-corrected correlations (PET-PEESE; Borenstein et al., 2021; Stanley & Doucouliagos, 2014).

Initially, our plan included examining the moderating effects of reliability, gender, and age (see https://osf.io/4z6s9). However, challenges arose due to data limitations. Many studies used only one or two items per Big Five trait and did not report reliability indices. This lack of data reduced our sample to just three studies for the reliability analysis, leading to inflated standard errors and the subsequent decision to exclude this moderator from the meta-analysis. We encountered similar problems with gender and age, where the available data narrowed down to only 10 and 8 studies, respectively. This significant reduction in sample size also resulted in larger standard errors, rendering these analyses unfeasible in the meta-analytic framework. Nonetheless, the results from these preliminary estimations are available on OSF (https://osf.io/xjuqm).

### Results

**Figure 2.**
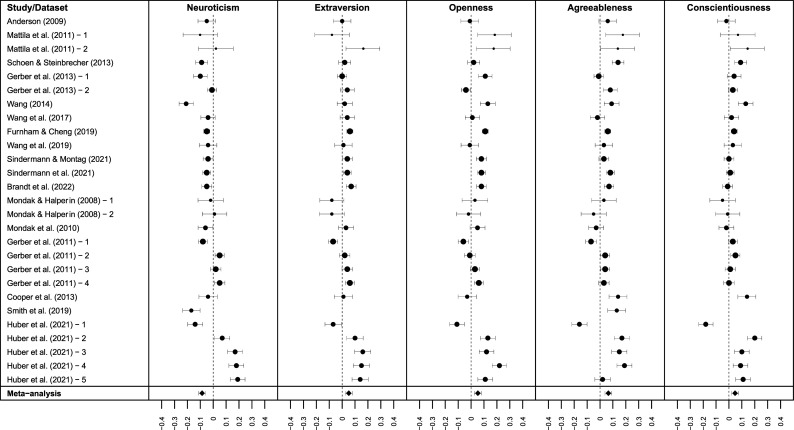
Forest plots depicting meta-analytic correlations with 95% confidence intervals. Larger point sizes indicate greater meta-analytic weights.

**Figure 3.**
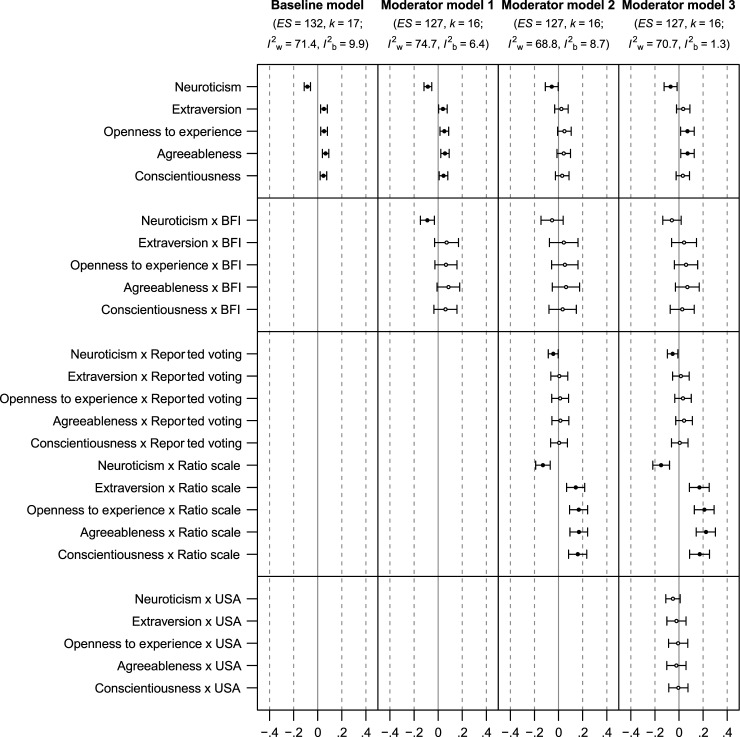
Meta-analytic personality-voting correlations, with corrected 95% confidence intervals. *Note. ES* = Number of effect sizes; *k* = Number of studies; *I*^2^_w_ = Within-study heterogeneity; *I*^2^_b_ = Between-study heterogeneity. In order of complexity, the reference conditions of the moderator models are (1) the Ten Item Personality Inventory (Gosling et al., 2003), contrasted with the Big Five Inventory (John et al., 1991); (2) voting intent and use of a dichotomous scale, contrasted with reported voting and use of a ratio scale; and (3) samples from outside the U.S., contrasted with samples from the U.S.. In each line plot, the limits denote the corrected 95% confidence intervals. Black points indicate point estimates where the confidence interval excludes zero. Confidence intervals are corrected using a sandwich estimator for the standard errors and Satterthwaite approximation for the critical values (Satterthwaite, 1946; Tipton & Pustejovsky, 2015).

Figure 2 shows the forest plot of effect sizes from the studies considered in the meta-analysis. Table 3 shows the main effects of the meta-analytic correlations between the Big Five personality traits and voting, without considering the influence of moderators. Figure 3 illustrates the meta-analytic correlations between the Big Five personality traits and voting across various moderator models, including corrected 95% confidence intervals. Individual statistics, including 95% prediction intervals for the correlations, are accessible at OSF (https://osf.io/57kgd).

**Table 3.** Main effects of the meta-analytic and mega-analytic personality-voting correlations.

	Meta-analysis		Mega-analysis	
	*r*	95% CI	*r*	95% CI
Neuroticism	−.086	[−.112, −.060]	−.035	[−.044, −.025]
Extraversion	.052	[.024, .079]	.099	[.079, .119]
Openness to experience	.053	[.025, .080]	.105	[.084, .125]
Agreeableness	.065	[.038, .092]	.100	[.080, .121]
Conscientiousness	.048	[.020, .075]	.093	[.072, .113]

*Note.* Number of effect sizes in meta-analysis = 132, *k*_Meta-analysis_ = 17, *I*^2^_Within_ = 71.4%, *I*^2^_Between_ = 9.9%; *N*_Mega-analysis_ = 41,077, *k*_Mega-analysis_ = 13.

#### Main effects of personality traits on voting outcomes

In the first model, which incorporated the Big Five as dummy-coded moderators, each trait showed a small yet significant correlation with voting intentions and behaviors in the hypothesized directions. Neuroticism had a negative correlation (*r* = −.09, 95% CI [−.11, −.06]) while extraversion (*r* = .05, 95% CI [.02, .08]), openness (*r* = .05, 95% CI [.03, .08]), agreeableness (*r* = .07, 95% CI [.04, .09]), and conscientiousness (*r* = .05, 95% CI [.02, .07]) each had positive correlations. Overall, and when not considering additional moderators, these findings indicate that Big Five personality traits are related to voting.

#### Brief versus longer measures

In the second model, we compared the number of items used to measure the Big Five with reference levels set at 2 items and 9 items. These benchmarks align with the item counts in the most frequently used brief personality inventories: TIPI (2 items per trait) and BFI (approximately 9 items per trait). Contrary to our hypothesis, longer measures resulted in wider standard errors and broader confidence and prediction intervals. Whereas the Big Five remained significantly correlated with voting outcomes using brief measures, this was not the case with longer measures. This counterintuitive finding may have to do with the tendency for studies with larger samples to use briefer measures, as we discuss below.

#### Voting intentions versus voting behaviors and dichotomous scales versus continuous scales

In the third model, we included voting conceptualization (intention/behavior) and operationalization (dichotomous/continuous) as dummy-coded moderators. As hypothesized, continuous scale measures of voting had stronger effect sizes (approximately Δ*r* = .05 to .10), and all Big Five traits demonstrated significant correlations with voting intentions. Specifically, neuroticism was negatively correlated (*r* = −.13, 95% CI [−.19, −.07]), while extraversion (*r* = .14, 95% CI [.07, .22]), openness (*r* = .17, 95% CI [.09, .24]), agreeableness (*r* = .17, 95% CI [.09, .24]), and conscientiousness (*r* = .16, 95% CI [.08, .23]) showed positive correlations.

Our hypothesis that the effect sizes would vary based on voting conceptualization was not supported; both intention-based and behavior-based measures yielded similar and nonsignificant results in studies that used dichotomous voting scales. The only exception was neuroticism, which showed significant correlations with both dichotomous voting intentions (*r* = −.06, 95% CI [−.11, −.00]) and with dichotomous voting behavior (*r* = −.04, 95% CI [−.09, −.00]), albeit without a notable difference between them. Overall, these results suggested that the observed correlations between the Big Five personality traits and voting outcomes are particularly pronounced with continuous intention measures, apart from neuroticism, which also correlated negatively in dichotomous and behavior measures.

#### Samples from the United States versus samples from other countries

Finally, in the fourth model, we included geographic location (U.S./other) as a dummy-coded moderator. While we did not preregister hypotheses, we found that the effects were more pronounced in countries outside the U.S., while those within the U.S. predominantly yielded nonsignificant results. This trend was apparent in dichotomous intention measures, where significant correlations were observed for neuroticism (*r* = −.07, 95% CI [−.12, −.02]), openness (*r* = .07, 95% CI [.01, .13]), and agreeableness (*r* = −.07, 95% CI [.02, .13]). The negative correlation of neuroticism with voting behavior was similar to previous models (*r* = −.05, 95% CI [−.10, −.01]).

Again, we found the strongest effects for continuous measures, with another notable difference coming from non-U.S. samples (approximately Δr = .02 to .05). Overall, this model suggested that correlations between the Big Five traits and voting outcomes are notably stronger in non-U.S. samples, with effects in the U.S. being nonsignificant.

## Mega-analysis

Following the conclusion of the meta-analysis, we revisited the literature identified during the systematic review to identify studies and datasets suitable for inclusion in the mega-analysis. After the individual data retrieval and harmonization, we conducted the mega-analysis and present the outcomes of this phase in the following sections.

### Individual data retrieval and harmonization

Of the studies identified in the systematic review, several were derived from publicly accessible data, and were thus retrievable for a mega-analysis. Table 1 includes a column labeled “Data” that lists these 14 datasets, spread across 13 studies. Of these 14 datasets, nine originated from established panel studies using comprehensive sampling techniques, whereas the remaining five were convenience samples tailored to the specific goals of each study. We initially planned to incorporate data from three additional panels identified in the systematic review: The 1992 Jyväskylä Longitudinal Study of Personality and Social Development (JYLS; Mattila et al., 2011), the 2012 Korean General Social Survey (KGSS; Wang et al., 2017), and the 2006 Cooperative Congressional Election Study (CCES; Mondak et al., 2010). Unfortunately, despite extensive efforts, access to these datasets was not feasible, resulting in 14 datasets used for the mega-analysis.

Before pooling the data, the various instruments used to measure personality and voting were harmonized by the first author. The detailed R script for this process is accessible on OSF (https://osf.io/v7j69). For the Big Five personality measures, we computed the trait scores independently rather than using the provided scores. This approach was feasible in every case except for the German Longitudinal Election Study (GLES; Schoen & Steinbrecher, 2013), which utilized the one-item-per-trait TIPI measure (Gosling et al., 2003). Missing values in any item were excluded in a pairwise manner, and scores indicating emotional stability were inverted to reflect neuroticism. Subsequently, all Big Five scores were converted to “Percentages Of the Maximum Possible” scores (POMPs; Cohen et al., 1999). POMPs are advantageous over other standardization methods, as they retain crucial data characteristics like mean, variance, skewness, and kurtosis. They are calculated as the ratio of a score’s deviation from the minimum to the range between maximum and minimum possible scores. No further weighting, for instance based on questionnaire length, was applied to the personality scores, as that variable was intended for use as a moderator later and should not be incorporated twice.

Voting measures that were not already dichotomized were transformed into dichotomous variables, given that most measures in the dataset were dichotomous. Each non-dichotomous voting variable was assessed for its content and distribution to decide which scale points should be used as the poles of the new dichotomous variable. This dichotomization was carried out in five datasets. In The American National Election Studies (ANES; Wang, 2014) and in study samples 1 (Gerber et al., 2013) and 5 (Smith et al., 2019), where voting intention was initially measured on equidistant five-, ten-, or one-hundred-point scales, all scores exceeding 75% voting intention were recoded as one (“yes”) and all scores at 75% or below were recoded as zero (“no”). In the Cooperative Campaign Analysis Project (CCAP; Gerber et al., 2011), voting behavior was aggregated over elections in 2000, 2002, 2004, and 2005, resulting in a five-point scale ranging from 0 (individuals who never voted) to 4 (individuals who voted in every election). A score of one (“yes”) was assigned to those with a total of one or higher (indicating participation in at least one election), while a score of zero (“no”) was assigned to those with a total of zero (indicating no participation). In the Minnesota Twins Political Survey (MTPS; Verhulst, 2012), voting intention was measured on a four-point scale (0 = “definitely will not vote”, 1 = “probably will not vote”, 2 = “probably will vote”, 3 = “definitely will vote”), and the scores were split so that an inclination toward voting was coded as one (“yes”) and not voting as zero (“no”).

Additionally, sociodemographic variables were standardized. The gender variable was harmonized to include only male and female categories, and the nationality variable was adjusted to distinguish between samples from the U.S. and those from other countries for consistency with the meta-analysis. Finally, data from the various studies were pooled into a single, combined dataset. In this process, any potential sampling weights, such as those found in the Cooperative Campaign Analysis Project (CCAP; Gerber et al., 2011) or the AmericasBarometer (AB; Weinschenk, 2013) were disregarded due to the impossibility of extending them to other samples. Hence, in these instances, the entirety of the available data was used.

### Summary of the literature

Table 2 summarizes key statistics for the 13 studies included in the mega-analysis. These studies included 14 datasets (e.g., Gerber et al., 2011; Gerber et al., 2013; Mattila et al., 2011).

The average sample size across individual studies in the mega-analysis was 3,158 participants (*SD* = 2,192, *Range* = 800–9,790), nearly double the average sample size observed in the meta-analysis. Gender distribution remained balanced in most studies, with 54.8% women on average, and a maximum of 61.6% in Brandt et al. (2022). Participants were again mostly middle-aged adults, with an average age of 46.2 years, and a minimum age of 23.0 years in one study (Weinschenk et al., 2019). Similar to the meta-analysis, the majority of studies used scales with one or two items per trait (i.e., the FIPI or TIPI; Gosling et al., 2003) or the BFI (eight to ten items per trait; John et al., 1991) to assess the Big Five personality traits. The distributions of voting conceptualization (intention/behavior), operationalization (dichotomous/continuous), and geographic location (U.S./other) were relatively balanced in the mega-analysis. Outside the United States, datasets were sourced from various countries, including Germany (Brandt et al., 2022; Schoen & Steinbrecher, 2013; Sindermann et al., 2021; Sindermann & Montag, 2023; Weinschenk et al., 2019), Great Britain (Furnham & Cheng, 2019), and Spain (Gallego & Oberski, 2012). However, the data in the mega-analysis were somewhat less culturally diverse compared to the meta-analysis, which included a broader range of East Asian countries.

### Analysis plan

In order to mirror the three-level structure employed for the meta-analysis as closely as possible, we tested hierarchical linear models (Burke et al., 2017; Curran & Hussong, 2009). Our approach entailed the inclusion of random intercepts to capture the inherent variability at the individual and study levels, acknowledging that individual responses are nested within broader study contexts. Additionally, to address the variability associated with the Big Five personality traits across studies, we incorporated random intercepts for each trait within studies. This modeling decision allowed us to account for the fact that both the baseline levels of voting outcomes and the effects of personality traits on these outcomes could exhibit variability across different study settings. The basic form of the model was as follows; personality scores were centered at zero:

$$\text{Level}\;1\;\left(\text{within}\;\text{participants}\right):{votingScore}_{ijk}=\beta_{0j}+\beta_{1j}\times {personalityScore}_{ijk}+\beta_{2jk}\times {personalityTrait}_{k}+\beta_{3jk}\times {personalityScore}_{ijk}\times {personalityTrait}_{k}+\varepsilon_{ijk}$$

where voting Score_ijk_ is the voting score for the i-th participant in the j-th study associated with personality trait k; β_0j_ is the intercept for study j, adjusted for each study; β_1j_ is the slope for the personality score within study j, reflecting how it affects the voting score within that study; β_2jk_ represents the effect of the k-th personality trait within study j, assuming the Big Five score is 0; β_3jk_ is the interaction effect within study j between the Big Five score and the k-th Big Five trait; and ε_ijk_ is the residual error for each observation.

$$\text{Level}\;2\;\left(\text{within}\;\text{studies}\right):\beta_{0j}=\gamma_{00}+u_{0j};\;\beta_{1j}=\gamma_{10};\;\beta_{2jk}=\gamma_{20k}+u_{2jk}$$

where γ_00_ is the overall average intercept across all studies; u_0j_ is the random effect capturing variability in the intercepts across studies; γ_10_ is the overall average slope for the Big Five score across all studies, assumed to be fixed in this simplification; γ_20k_ is the average effect of the k-th Big Five trait across all studies; and u_2jk_ is the random effect capturing variability in the effects of Big Five traits across studies.

$$\text{Level}\;3\;\left(\text{across}\;\text{studies}\right):u_{0j}∼N\left(0,\sigma_{u0}^{2}\right){;\;u}_{2jk}∼N\left(0,\sigma_{u2k}^{2}\right)$$

where σ_u0_^2^ is the variance of the random intercepts across studies and σ_u2k_^2^ is the variance of the random slopes for the personality traits across studies.

Then, we fitted a series of mixed-effects binomial logistic regression models to the data, closely replicating the meta-analytic procedure. The initial model was designed to examine how the Big Five personality traits correlate with voting outcomes. Each of the Big Five traits was represented by a dummy variable (0 or 1), indicating the presence of that trait, which, when interacted with the trait’s continuous score, served as the independent variable in predicting the dependent variable of voting outcomes. We then progressively included additional moderators in the analysis: (1) the number of items used for personality measurement (brief/longer), (2) the conceptualization and operationalization of voting (intention/behavior, and dichotomous versus continuous scaling), and (3) sociodemographic variables (nationality, gender, age). Ultimately, we also converted the model statistics into correlations and reported point estimates, along with 95% confidence intervals (Borenstein et al., 2021).

As with the meta-analyses, our mega-analytic procedure was preregistered and based on a limited set of a priori hypotheses, and thus we did not apply formal multiple-comparison corrections (e.g., Bonferroni adjustments).

### Results

**Figure 4.**
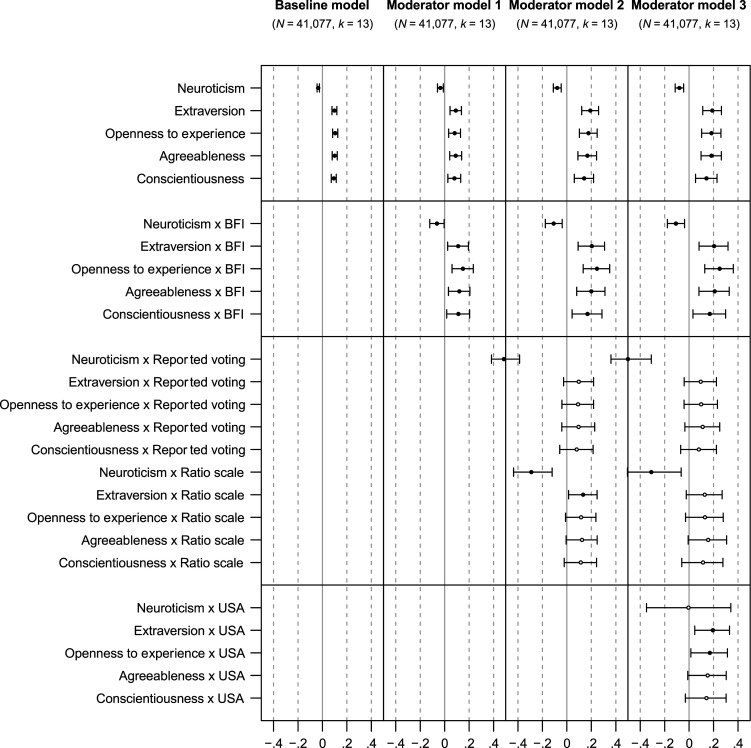
Mega-analytic personality-voting correlations, with 95% confidence intervals. *Note. N* = Number of individuals; *k* = Number of studies. In order of complexity, the reference conditions of the moderator models are (1) the Ten Item Personality Inventory (Gosling et al., 2003), contrasted with the Big Five Inventory (John et al., 1991); (2) voting intent and use of a dichotomous scale, contrasted with reported voting and use of a ratio scale; and (3) samples from outside the U.S., contrasted with samples from the U.S.. In each line plot, the limits denote the 95% confidence intervals. Black points indicate point estimates where the confidence interval excludes zero.

Table 3 shows the main effects of the mega-analytic correlations between the Big Five personality traits and voting, without considering the influence of moderators. Figure 4 illustrates the mega-analytic correlations between the Big Five personality traits and voting across various moderator models. 95% confidence intervals for these correlations are accessible on OSF (https://osf.io/57kgd).

#### Main effects of personality traits on voting outcomes

The first model incorporated only a regression of the voting score on the interaction of the dummy-coded Big Five personality traits and the personality score. Each trait showed a small yet significant correlation with voting outcomes in the hypothesized directions. Neuroticism had a negative correlation (*r* = −.03, 95% CI [−.04, −.03]) while extraversion (*r* = .10, 95% CI [.08, .12]), openness (*r* = .10, 95% CI [.08, .13]), agreeableness (*r* = .10, 95% CI [.08, .12]), and conscientiousness (*r* = .09, 95% CI [.07, .11]) each had positive correlations. Compared with the meta-analysis, the confidence intervals were narrower, likely due to increased statistical power. Also, except for neuroticism, the observed effects were slightly more pronounced. The larger and more precise effect sizes may have to do with the increased statistical power and the application of uniform statistical models which reduce the variability introduced by different analytical techniques, study designs, and measurement models that may have weakened the meta-analytic effects. Overall, however, the present findings confirm the meta-analytic results, implying that the Big Five personality traits have small yet significant correlations with voting outcomes.

#### Brief versus longer measures

In the second model, we additionally compared the number of items used to measure personality traits with reference levels set at 2 items and 9 items. These benchmarks align with the item counts in the most frequently used brief personality inventories: TIPI (2 items per trait) and BFI (approximately 9 items per trait). Similar to the meta-analytic findings, and, again, contrary to our hypothesis, longer measures resulted in wider standard errors and broader confidence and prediction intervals. In contrast to the meta-analysis, however, effects based on longer measures were still significantly correlated with voting outcomes, presumably due to higher power. These effects were also slightly larger than the effects based on the briefer measures (approximately Δ*r* = .02 in both directions). Specifically, neuroticism was negatively correlated (*r* = −.06, 95% CI [−.12, −.00]), while extraversion (*r* = .11, 95% CI [.02, .19]), openness (*r* = .15, 95% CI [.06, .23]), agreeableness (*r* = .12, 95% CI [.03, .21]), and conscientiousness (*r* = .11, 95% CI [.02, .20]) showed positive correlations.

Overall, these findings challenge the meta-analytic conclusion that the correlations observed are somewhat specific to shorter scales, although they corroborate that longer scales do not go hand in hand with smaller standard errors (Heene et al., 2014).

#### Voting intentions versus voting behaviors and dichotomous scales versus continuous scales

In the third model, we additionally compared different voting conceptualizations (intention/behavior) and voting operationalizations (dichotomous/continuous scales). The findings generally reflected the patterns noted in our meta-analysis, albeit with distinct variations. We observed a significant negative correlation between neuroticism and dichotomous measures of voting behavior (*r* = −.52, 95% CI [−.62, −.39]), as well as with continuous assessments of voting intentions (*r* = −.29, 95% CI [−.44, −.12]). Additionally, there was a significant positive correlation between extraversion and continuously assessed voting intentions (*r* = .13, 95% CI [.01, .25]). Diverging from the meta-analytic findings, we found no significant correlations between openness to experience, agreeableness, and conscientiousness, and continuous scale voting intentions. However, as before, the correlations for dichotomous voting intentions remained significant across both brief and long scales, with effect sizes consistently stronger than those in the previous models (approximately Δ*r* = .05 to .10).

These results question the meta-analytic conclusion that continuous scales invariably yield larger effects. In the mega-analysis, larger effects were only evident for neuroticism and extraversion, hinting that these effects are not robust across traits. The significant negative correlation for neuroticism, also evident in behavior-based measures, aligned with the meta-analytic results.

#### Samples from the United States versus samples from other countries

In our fourth model, we further differentiated between samples originating from the United States and those from other countries. Again, the findings here aligned with those of the meta-analysis in some respects but differed in others. We observed that all effects remained significant in non-U.S. samples, whereas in the meta-analysis, significance was only observed for neuroticism, openness, and agreeableness. Also, in U.S. samples, significant correlations emerged for extraversion (*r* = .19, 95% CI [.05, .33]) and openness to experience (*r* = .17, 95% CI [.01, .31]). However, continuous-scale based voting intentions did not yield significant correlations, with the exception of neuroticism. This trait continued to demonstrate a negative correlation with dichotomous voting behaviors, consistent with the previous model. Similar to previous models, the correlations for dichotomous voting intentions were also significant across both brief and longer scales.

These results contrast with the meta-analytic conclusion that the links between the Big Five personality traits and voting outcomes are predominantly relevant to non-U.S. samples. In our mega-analysis, we identified similarly sized effects for extraversion and neuroticism in U.S. samples, suggesting that such effects might again be trait-specific.

#### Effects of gender and age

While this was not possible in the meta-analysis due to missing data, we examined the moderating effects of gender and age in the mega-analysis. The results of these analyses, including point estimates and 95% confidence intervals, are available on OSF (https://osf.io/57kgd).

In our fifth model, we differentiated between men and women. The analysis revealed that the influence of all Big Five personality traits remained consistent in both directions and significance for both genders, indicating no significant gender-based differences in their effects on voting behavior.

In our sixth model, we differentiated between participants aged 25 years, 50 years, and 75 years. Similar to the gender analysis, the direction and significance of the effects was largely robust across these age categories. The sole exception was conscientiousness, which was not a significant predictor across all age groups. In these analyses, the standard errors expanded substantially, underscoring that even the robust dataset of the mega-analysis was insufficient to yield reliable estimates for these effects.

## Discussion

Voting plays a critical role in the vitality and development of democratic systems, with effectiveness relying heavily on active voter involvement (Flanagan & Levine, 2010; Metzger et al., 2018). Personality traits have been identified as important predictors of voting, yet previous findings were inconclusive, with no single personality trait consistently linked to voting intentions and behaviors (e.g., Brandt et al., 2022; Furnham & Cheng, 2019; Gerber et al., 2013; Weinschenk & Dawes, 2018). This study used data from a systematic review to provide robust estimates of the links between personality traits and voting behaviors by integrating the results of meta-analysis and mega-analysis.

Across both types of analysis, we found modest but consistent links between the Big Five personality traits and voting. Specifically, individuals with favorable voting intentions tend to be higher in extraversion, openness to experience, agreeableness, conscientiousness, and lower in neuroticism. Low neuroticism emerged as the only trait predicting both voting intentions and behavior, underscoring its importance. The null correlations between the other Big Five and specific voting behaviors is consistent with previous research on personality traits and civic behavior (e.g., Bleidorn, Stahlmann, Orth, et al., 2025; Soutter et al., 2020) and points to the relevance of current, situational factors for individual voter engagement in single elections. Contrary to our hypotheses, longer measures did not result in smaller standard errors, and continuous measures of voting did not yield larger effect sizes. Effects were somewhat more pronounced outside the U.S., but age and gender did not account for notable differences.

We can draw four main implications from these findings. First, the Big Five traits are reliable predictors of voting intentions, but there is a need for well-powered studies to elucidate their impact on concrete civic actions like voting outcomes. Second, the moderators we examined in this study did not significantly alter the findings, so exploring others may prove particularly beneficial. Third, neuroticism stands out as a noteworthy personality trait in understanding voting behaviors, suggesting the need for additional research attention. Fourth, the present results hold promise for informing interventions designed to encourage voting participation and protect democratic processes against undue influence. We now discuss the scope and significance of these implications.

### Importance of integrative data analysis, longer personality measures, and modest effects

Our integrative data analysis suggests that previous studies have lacked the necessary statistical power to consistently capture the modest yet significant associations between personality traits and voting outcomes. In the meta-analysis, the range of prediction intervals often spanned from negative to null to positive effect sizes, reflecting the variability and inconsistencies found within the literature. In the mega-analysis, the addition of multiple moderators rapidly resulted in increased standard errors, an issue that is likely to be even more pronounced in individual studies with limited statistical power. Only the negative correlation between neuroticism and ratio-based measures of voting intentions and turnout proved strong and robust enough to potentially be detected in individual studies with substantial power. In other cases, our analysis cautions against overreliance on single-study outcomes, as they may mislead rather than illuminate the nuanced links between personality traits and civic actions like voting (Hedges & Tipton, 2010; Hunter & Schmidt, 1982).

To find robust effects, future research might consider consistently incorporating comprehensive personality measures in extensive data collection efforts. Our results indicated that the use of longer personality measures did not necessarily lead to more consistent results. However, this observation may stem from a limited dataset, where the prevalent approach in existing literature typically matches large samples with concise personality scales, or smaller samples with in-depth measures (Credé et al., 2012; Sleep et al., 2021; Soto & John, 2019). In our meta-analysis, studies utilizing longer measures were assigned lesser weights due to their smaller sample sizes, compared to those with larger samples but brief measures. Overall, the systematic review did not yield any studies that included highly comprehensive personality measures, except for the study by Mattila et al. (2011), which could not be considered in the mega-analysis. To evaluate the role of different personality trait scales, a systematic incorporation of longer measures into large-scale studies is necessary (e.g., Haehner, Krämer, et al., 2025). Beyond the potential for more consistent results, longer measures can offer superior construct coverage, greater variance, reduced measurement error (via latent variable modeling), and more finely grained descriptions (e.g., via facet-level analysis), as will be discussed further (Anglim & O’Connor, 2019; Sleep et al., 2021; Smith et al., 2000).

Despite their modest size and the challenge of robustly estimating them in single studies, it is important to highlight the significant influence that correlations between personality traits and voting can wield when aggregated across populations (Funder & Ozer, 2019; Ozer & Benet-Martínez, 2006). Previously, we illustrated how even odds ratios of 1.1 could sway tightly contested elections if leveraged to their potential. However, in light of our findings, these numbers were underestimated. If we rerun our calculation with assumed odds ratios between 1.2 and 1.4, depending on whether we look at the meta-analysis or the mega-analysis, we can estimate that a political campaign with 5% success rate would yield an estimated 692,000 additional votes when targeting individuals high in traits like extraversion, conscientiousness, agreeableness, and openness.^6^ Conversely, targeting a group low in these traits might only yield 527,000 additional votes.^7^ This means that, when targeting people with personalities more inclined to vote instead of those less inclined to vote, up to 165,000 additional votes may be secured, marking a significant difference of 31.3% in favor of effective personality targeting. This calculation demonstrates the importance of not underestimating or disregarding these seemingly modest effects in political campaigning or similar endeavors that could leverage Big Five profiling.

### Differences in domain-coverage and between lower-order traits

We found no moderation effects of sociodemographic factors such as gender and age, and minimal evidence for moderation effects of the sampling region and the operationalization of voting measures as either dichotomous or continuous measure. The only notable sociodemographic effect was that associations were somewhat more pronounced outside the U.S. This may reflect contextual variation in how personality translates into political behavior. For instance, personality–participation links may differ across countries due to variation in political norms, institutional rigidity, and the openness of civic spaces (Weinschenk, 2017). Personality may play a larger role where political engagement is less tightly structured by external rules or expectations.

Other than that, the persistent heterogeneity observed both within and across the studies in the meta-analysis suggests the possibility of other moderators not examined in this research. These other moderators, either alone or in combination, might explain additional differences in the correlation between personality traits and voting. Some of these differences may be partly attributed to the inherent variability in how traits are conceptualized and measured across questionnaires and test families (Miller et al., 2011). Such variability arises because lower-order components, including aspects, facets, or nuances, are represented and emphasized differently in each questionnaire (Crowe et al., 2018; Mõttus et al., 2017; Schwaba et al., 2020). For instance, some measures of neuroticism might capture more content related to heightened anxiety and withdrawal tendencies, while others might capture more content related to anger and volatility. This discrepancy poses challenges, particularly when hypotheses formulated based on one model of the Big Five are tested using other questionnaires with slightly different conceptualizations of these traits. Therefore, alongside questionnaire length, the conceptual distinctions within the Big Five, such as between questionnaires like the TIPI and BFI, warrant further exploration as potential moderators in future research.

Moreover, while facets within broader personality trait dimensions are highly correlated, their predictive validity may differ across outcomes and situations. For example, although a high level of neuroticism typically heightens stress sensitivity, the related lower-level facets of anxiety and anger may produce contrasting effects on voting. Anxiety may discourage voter participation due to the stress associated with political debates (Gerber et al., 2013; Schoen & Steinbrecher, 2013), whereas anger may motivate individuals to participate in voting as a means of protest or alleviation (Brader, 2005; Marcus & MacKuen, 1993). There is a growing body of research supporting the idea that considering both domain-level traits and their sub-components can resolve inconsistencies and improve the accuracy of predictions (Anglim et al., 2019; Mõttus, 2016; Stahlmann et al., 2023). However, the literature in our systematic review did not use such detailed personality assessments. Therefore, employing more comprehensive personality questionnaires, which provide scores for more domains and specific facets, could elucidate effects that appear contradictory at the domain level but are coherent at the facet level.

Finally, we approached this project with the idea that overarching moderators might govern the correlations between Big Five traits and voting outcomes. For example, our initial prediction was that these associations would be strongest for voting attitudes measured on continuous scales among middle-aged adults (35–64 years), with uniform effects across all traits. However, some moderators may affect only specific traits or even counteract one another, leading to null average effects. Thus, more sensitive predictions that focus on specific trait-moderator interactions are warranted. One promising strategy is specification curve analysis—a method that aggregates and disaggregates results across various moderators (Simonsohn et al., 2020). In the context civic engagement, this approach revealed that extraverted younger adults were less likely to register as organ donors, whereas extraverted older adults were more likely (Stahlmann et al., 2023). However, given the reduced sample sizes in specific personality–moderator subgroups when multiple moderators are considered, these findings should be interpreted with caution. Therefore, integrating specification curve analysis with meta-analysis would thus provide a rigorous means to assess the stability of such nuanced effects (Voracek et al., 2019).

### Neuroticism’s role in voting behavior and its broader societal effects

The personality trait that stood out the most in our analysis was neuroticism, which not only diminished the intent to vote but also actual participation in voting. This finding complements previous research demonstrating the personal and societal costs of higher neuroticism. For instance, neuroticism is associated with increased healthcare costs (Cuijpers et al., 2010; Lahey, 2009; Widiger & Oltmanns, 2017) and a wide range of adverse health outcomes, including unhealthy habits (e.g., smoking and excessive alcohol consumption), chronic physical conditions, mental health issues, and higher mortality rates (Bleidorn, Stahlmann, & Hopwood, 2025; Charles et al., 2008; Jokela et al., 2014; Kotov et al., 2010; Malouff et al., 2007; Terracciano & Costa, 2004). Altogether, the societal costs of neuroticism are estimated as more than twice as high as the costs of common mental disorders (Cuijpers et al., 2010).

In light of the significant repercussions associated with neuroticism, the strategy of diminishing its impact through specific interventions has attracted growing interest. Altering a personality trait like neuroticism may seem unconventional, yet the desire to improve personal traits is common throughout the general population (Hudson & Fraley, 2015; Hudson & Roberts, 2014; Thielmann & de Vries, 2021). Evidence suggests that psychotherapy can effectively reduce neuroticism levels (Armstrong & Rimes, 2016; Nyklíček & Irrmischer, 2017; Roberts et al., 2017; Wright et al., 2024). However, it is still a matter of some debate whether such changes are sustained over the long term, as opposed to being mere temporary alleviations of symptoms or other difficulties. Furthermore, research indicates that purposeful personality change is possible within the general population, particularly for those who are driven to change, believe in the possibility of change, and engage in new practices that lead to personality adjustments (Allemand & Flückiger, 2022; Haehner et al., 2024; Olaru et al., 2022; Stieger et al., 2018). These insights collectively suggest the potential for volitional personality change beyond natural maturation, although the precise drivers, incentives, and moderating factors of such transformations are yet to be comprehensively understood.

Addressing the 10.68% drop in U.S. voter turnout over the past six decades (Coppedge et al., 2023) may well lie in the realm of personality psychology, particularly through interventions aimed at reducing neuroticism. Our findings show that people scoring one standard deviation above the mean on neuroticism are less likely to vote, with odds ratios between 1.4 and 4.9, depending on whether we look at the meta-analysis or the mega-analysis. With about 80 million of the 240 million eligible voters in the U.S. motivated to increase their emotional stability, the implications of such personal growth are significant (see Thielmann & de Vries, 2021). If we assume that 1% of these motivated individuals, or 800,000 people, successfully decrease their neuroticism by one standard deviation, the adjusted voting probabilities for this group could settle in between 68.65%^8^ and 88.46%,^9^ compared to the current average of 61%. These heightened probabilities would lead to an additional 61,200^10^ to 219,6800^11^ votes. Such findings not only highlight the significance of neuroticism on electoral participation but also suggest that interventions aimed at reducing it could play a pivotal role in mobilizing voters, especially in closely contested elections.

Future research should test whether personality interventions lead to lasting changes in political behavior. Longitudinal and cross-national studies are needed to assess how robust and generalizable such effects are. Large panel studies, such as the Longitudinal Internet Studies for the Social Sciences (LISS; Scherpenzeel & Das, 2011) and the Understanding America Study (UAS; Alattar et al., 2018), provide infrastructure for tracking personality and civic engagement over time. In contrast to these observational designs, the CHILL study (Haehner, Wright, et al., 2025) targets neuroticism through structured training and includes multi-informant assessments and repeated follow-ups across German-speaking countries. While it does not measure political behavior, CHILL and similar studies can offer a model for studying sustained personality change and may serve as a blueprint for future research linking such change to political participation.

### Implications for the role of personality psychology in politics

While the potential to enhance voter participation by addressing neuroticism is promising, our findings also highlight risks, particularly the possibility of misuse by unscrupulous actors aiming to manipulate political outcomes. The Cambridge Analytica controversy, for example, has brought to light the potentially impactful role of personality psychology in the political arena. Although the precise effect of Cambridge Analytica’s strategies on the 2016 U.S. presidential election remains debated (Gibney, 2018; Grassegger & Krogerus, 2017), there is an accumulating body of evidence showing that strategies employing personality psychology can indeed foster more favorable attitudes toward specific political parties or candidates (Kosinski et al., 2015; Matz et al., 2017).

The underlying process of these strategies involves the initial assessment of personality traits, followed by the alignment of messages that resonate with the identified personality profiles (Matz et al., 2023). These messages are often combined with imagery or details about a party’s program or a candidate, reminiscent of classical conditioning techniques. For instance, individuals characterized by high levels of neuroticism might encounter political ads that highlight threats or fears, intertwined with topics such as warfare, criminal activity, or immigration issues, which a party aims to address (Caprara & Zimbardo, 2004; Krotzek, 2019). This strategy of personality assessment followed by targeted political messaging has proven effective within social media platforms, where personality data can be gleaned from biographical texts and political ads are integrated into users’ timelines (Zarouali et al., 2022).

Understanding how the results of our analyses tie into this line of research could help in developing strategies to guard against undue influence. For example, our findings suggest that focusing on individuals with high neuroticism for political targeting may be ineffective, as their likelihood to vote is lower despite potential support for a political party. Instead, directing efforts toward individuals with lower neuroticism and customizing messages to their other traits, like extraversion or agreeableness, may become a preferred strategy for organizations seeking to influence voter allegiances. Consequently, it might be prudent to adjust preventive efforts toward individuals who are emotionally stable, as they are more likely to vote and thus may be more exposed to persuasive political content.

This illustrative example underscores the ethical dilemma of our research findings: Psychological targeting, particularly without individuals’ awareness, may threaten autonomy and informed choice (Matz et al., 2017). Yet such strategies are already used by political actors and tech firms, often without ethical oversight (Matz & Netzer, 2017). For this reason, some have argued that psychology has an ethical obligation to engage with these developments in order to help guide responsible use (Matz et al., 2023). Rather than dismissing this line of research as inherently unethical, it may be more prudent to stay one step ahead—so that potential misuse can be identified and prevented early on.

### Strengths and limitations

This study leverages both meta-analysis and mega-analysis to offer a detailed and robust examination of the link between the Big Five personality traits and voting outcomes. By systematically reviewing data from both psychological and broader academic sources via Scopus and the grey literature database BASE, we compiled information from 65,036 individuals across 17 studies for the meta-analysis, and from 44,206 individuals across 13 studies for the mega-analysis. Our integrative analysis encompassed zero-order correlations and explored how nationality, gender, age, and the specific measures of personality and voting outcomes might moderate these correlations. Despite these strengths, the study faces certain limitations.

#### Self-report data and potential for inflated correlations

The personality and voting measures considered in this research were based on self-reporting, which have faced scrutiny for their susceptibility to social desirability bias and the potential for inflated correlations due to common method bias. Social desirability refers to the extent to which something is viewed favorably, affecting not only personality traits but also self-reported behaviors such as voting (Holden, 2010). There is a concern that individuals might misrepresent themselves, either intentionally or subconsciously, to maintain a certain self-image or to appear favorable in the eyes of others. Traditional approaches to mitigate this issue included the use of “lie scales” and evaluating self-other agreement to assess the tendency to distort responses or modeling social desirability as a distinct factor to isolate the “true” correlation between personality traits and behaviors like voting (Larson, 2019; Nederhof, 1985). However, evaluative elements are often integral to personality traits, and attempting to separate descriptive from evaluative components might risk stripping them of part of their defining content (Konstabel et al., 2006; Ones et al., 1996). Furthermore, targeted investigations into the presumed drawbacks of self-reports often yield encouraging results, affirming their agreement with other-reports and robustness to social desirability bias (Howard et al., 1980; Kim et al., 2019). Considering the data in this study were gathered in low-stakes environments, with participants having little motivation to skew their answers, we find scant basis for broadly questioning the validity of the self-report data and the conclusions drawn from them.

The issue of common method bias, however, is a valid concern in our study. This bias occurs when the same measurement method, such as self-report, is used for both predictors and outcomes, which can inflate correlations (Conway & Lance, 2010). Estimates suggest this inflation averages around 25% in typical behavioral research contexts (Podsakoff et al., 2003). Adjusting for this inflation, our correlations (r ≈ .05–.10) realistically correspond to true values of approximately r ≈ .04–.08. Applying this correction to our voter-mobilization estimate (originally 165,000 additional votes), the adjusted figure would be approximately 124,000 votes. Similarly, estimated effects from neuroticism-reduction interventions (originally 61,200–219,680 votes) would be reduced to approximately 45,900–164,800 votes. Thus, common method bias may have inflated the correlations. However, the practical significance should remain important even when accounting for it.

Nevertheless, future research should endeavor to incorporate a variety of assessment methods, such as behavioral observations, physiological measures, or analysis of official voting records, in addition to self-and other-report measures. Also, an inclusion of a broader range of other, more specific personality constructs, such need for cognition (Arceneaux & Vander Wielen, 2013) or political interest (Denny & Doyle, 2008), may facilitate more precise and comprehensive insights into the connection between personality traits and voting outcomes. In general, we would expect that the more similar the content of a personality trait is to voting attitudes and behavior, the stronger its validity in this domain (Gerber et al., 2011).

#### Data harmonization and convergence issues

For our mega-analysis, we harmonized varied data in different formats and languages. This included establishing a standard data format, creating rules to merge voting information from diverse studies, and setting criteria to convert continuous variables into binary ones. While essential, this process inevitably introduced subjectivity. We documented our methods for transparency and replicability. Nonetheless, it is important to acknowledge that our data management and analysis choices have shaped our findings, and alternative strategies by other researchers might lead to nuanced differences in conclusions.

Additionally, we encountered challenges when fitting log-linear models to our data. Our goal was to mirror the random-effects structure of the meta-analysis, maintaining its three-level design. Yet, introducing multiple moderators occasionally led to model non-convergence. Attempts to simplify the random-effects structure in sensitivity analyses did not resolve the issue. We found that the fixed effects reported were largely consistent, regardless of adjustments to the random-effects structure, thus supporting their robustness. However, the estimates of random effects and variance components should be interpreted with caution, as they were more susceptible to changes in the model structure.

#### Missing sociodemographic data and WEIRD samples

Another key limitation is the incomplete sociodemographic data, which restricted our ability to examine gender and age as potential moderators in the meta-analysis. Information on race and ethnicity was also largely missing. While many samples were gender-balanced, some were strongly female-biased, and age distributions were skewed toward middle adulthood (roughly 22–60 years), with older adults being underrepresented. This limited variance complicates cross-demographic comparisons and may have reduced statistical power to detect moderation effects. For example, some studies in our review reported significant age effects that we could not replicate. Prior research suggests that the influence of personality on political behavior may vary by age and life stage (e.g., Mondak et al., 2010; Stahlmann et al., 2023). Whether such effects are absent at the aggregated level or obscured by sampling constraints remains unclear.

Moreover, the majority of studies we reviewed were conducted in Western, educated, industrialized, rich, and democratic (WEIRD) contexts, primarily in Northern Europe and North America (Henrich et al., 2010). We had limited representation from non-Western countries such as Brazil, Russia, Korea, and Taiwan, and no inclusion of African nations. This typical but ultimately narrow scope, combined with the lack of racial and ethnic data, means our findings are predominantly relevant to a WEIRD demographic. Additionally, our literature search was confined to English and German publications, which may overlook valuable insights from research conducted in other languages. To enhance the universality and specificity of our understanding of personality trait dynamics, further investigation into the cultural and ethnic nuances of the link between personality and voting is essential (Thalmayer et al., 2021).

### Conclusion

Our combined meta-analytic and mega-analytic approach has clarified the links between the Big Five personality traits and voting intentions and behavior, hitherto obscured by a fragmented and inconsistent literature. Our findings suggest that the Big Five are modest but significant predictors of voting intentions, with neuroticism additionally predicting voting behavior. To obtain reliable results, large sample sizes and extensive personality assessments are important, enabling a deeper understanding of the connection between personality and civic participation like voting. These insights may prove valuable for developing strategies to increase voter participation and protect against manipulative influences on the democratic process. Questions remain about the processes by which the observed links between personality traits and intentions may manifest into actual voting behaviors. A crucial next step for personality theory and research would thus be to document how personality traits may shape voting intentions and ultimately cause actual voter behavior.

## Notes

1. 20,000,000 × 61% × 5% = 610,000 (baseline voting probability from Coppedge et al., 2023).

2. 20,000,000 × ((61% × 1.10)/(1%–61% + (61% × 1.10))) × 5% = 632,400.

3. 20,000,000 × ((61% × (1/1.10))/(1%–61% + (61% × (1/1.10)))) × 5% = 587,700.

4. The BASE search engine lacks the functionality for advanced search term combinations. Consequently, when searching through grey literature, the original query was segmented into 31 discrete queries (see https://osf.io/x9pzh).

5. It is worth noting that the available data could potentially support a four-level model, where individual data points are nested in measures of different personality traits or voting conceptualizations/operationalizations, which are then nested in specific samples, and finally, in individual studies. Preliminary analysis, however, found that this added level did not explain any additional variation, leading us to proceed with a simpler three-level model. These preliminary analyses are available on OSF (https://osf.io/xjuqm).

6. 20,000,000 × ((61% × 1.44)/(1%–61% + (61% × 1.44))) × 5% = 692,000.

7. 20,000,000 × ((61% × (1/1.44))/(1%–61% + (61% × (1/1.44)))) × 5% = 527,000.

8. ((61% × 1.40)/(1%–61% + (61% × 1.40))) = 68.65%.

9. ((61% × 4.90)/(1%–61% + (61% × 4.90))) = 88.46%.

10. 68.65% * 800,000%–61% * 800,000 = 61,200.

11. 88.46% * 800,000%–61% * 800,000 = 219,680.

## References

[bibr1-08902070251383955] Adler R. P. Goggin J. (2005). What do we mean by “civic engagement”. Journal of Transformative Education, 3(3), 236–253. 10.1177/1541344605276792

[bibr2-08902070251383955] Alattar L. Messel M. Rogofsky D. (2018). An introduction to the Understanding America Study internet panel. SSRN Electronic Journal, 78(2), 13–28. 10.2139/ssrn.5226374

[bibr3-08902070251383955] Allemand M. Flückiger C. (2022). Personality change through digital-coaching interventions. Current Directions in Psychological Science, 31(1), 41–48. 10.1177/09637214211067782

[bibr4-08902070251383955] (1) (2) Anderson M. R. (2009). Beyond membership: A sense of community and political behavior. Political Behavior, 31(4), 603–627. 10.1007/s11109-009-9089-x

[bibr5-08902070251383955] Anglim J. Horwood S. Smillie L. D. Marrero R. J. Wood J. K. (2020). Predicting psychological and subjective well-being from personality: A meta-analysis. Psychological Bulletin, 146(4), 279–323. 10.1037/bul0000226 31944795

[bibr6-08902070251383955] Anglim J. O’Connor P. (2019). Measurement and research using the Big Five, HEXACO, and narrow traits: A primer for researchers and practitioners. Australian Journal of Psychology, 71(1), 16–25. 10.1111/ajpy.12202

[bibr181-08902070251383955] Appelbaum M. Cooper H. Kline R. B. Mayo-Wilson E. Nezu A. M. Rao S. M. (2018). Journal article reporting standards for quantitative research in psychology: The APA Publications and Communications Board task force report. American Psychologist, 73(1), 3–25. 10.1037/amp0000191 29345484

[bibr7-08902070251383955] Arceneaux K. Vander Wielen R. J. (2013). The effects of need for cognition and need for affect on partisan evaluations. Political Psychology, 34(1), 23–42. 10.1111/j.1467-9221.2012.00925.x

[bibr8-08902070251383955] Armstrong L. Rimes K. A. (2016). Mindfulness-based cognitive therapy for neuroticism (stress vulnerability): A pilot randomized study. Behavior Therapy, 47(3), 287–298. 10.1016/j.beth.2015.12.005 27157024

[bibr9-08902070251383955] Assink M. Wibbelink C. J. (2016). Fitting three-level meta-analytic models in R: A step-by-step tutorial. The Quantitative Methods for Psychology, 12(3), 154–174. 10.20982/tqmp.12.3.p154

[bibr10-08902070251383955] Barlow D. H. Ellard K. K. Sauer-Zavala S. Bullis J. R. Carl J. R. (2014). The origins of neuroticism. Perspectives on Psychological Science: A Journal of the Association for Psychological Science, 9(5), 481–496. 10.1177/1745691614544528 26186755

[bibr11-08902070251383955] Barrick M. R. Mount M. K. (1991). The Big Five personality dimensions and job performance: A meta-analysis. Personnel Psychology, 44(1), 1–26. 10.1111/j.1744-6570.1991.tb00688.x

[bibr12-08902070251383955] Bates D. Maechler M. Bolker B. Walker S. (2023). Lme4: Linear mixed-effects models using ‘Eigen’ and S4 (R package version 1.1-35.1). https://cran.r-project.org/package=lme4

[bibr14-08902070251383955] Beck E. D. Jackson J. J. (2022). A mega-analysis of personality prediction: Robustness and boundary conditions. Journal of Personality and Social Psychology, 122(3), 523–553. 10.1037/pspp0000386 35157487 PMC8867745

[bibr15-08902070251383955] Binder S. A. Smith S. S. (1997). Politics or principle? Filibustering in the United States senate. Rowman & Littlefield.

[bibr16-08902070251383955] Blais A. (2006). What affects voter turnout? Annual Review of Political Science, 9(1), 111–125. 10.1146/annurev.polisci.9.070204.105121

[bibr17-08902070251383955] Bleidorn W. Hill P. L. Back M. D. Denissen J. J. A. Hennecke M. Hopwood C. J. Jokela M. Kandler C. Lucas R. E. Luhmann M. Orth U. Wagner J. Wrzus C. Zimmermann J. Roberts B. (2019). The policy relevance of personality traits. American Psychologist, 74(9), 1056–1067. 10.1037/amp0000503 31829685

[bibr18-08902070251383955] Bleidorn W. Hopwood C. J. (2024). A motivational framework of personality development in late adulthood. Current Opinion in Psychology, 55(3), Article 101731. 10.1016/j.copsyc.2023.101731 38007918

[bibr19-08902070251383955] Bleidorn W. Stahlmann A. G. Hopwood C. J. (2025). Big Five personality traits and vaccination: A systematic review and meta-analysis. Health Psychology: Official Journal of the Division of Health Psychology, American Psychological Association, 44(1), 44–56. 10.1037/hea0001398 39298208

[bibr20-08902070251383955] Bleidorn W. Stahlmann A. G. Orth U. Smillie L. D. Hopwood C. J. (2025, March 3). Personality traits and traditional philanthropy: A systematic review and meta-analysis. 10.31234/osf.io/4sjhg_v1 40244970

[bibr21-08902070251383955] Borenstein M. Hedges L. V. Higgins J. P. Rothstein H. R. (2021). Introduction to meta-analysis. John Wiley & Sons.

[bibr13-08902070251383955] Bosco F. A. Aguinis H. Singh K. Field J. G. Pierce C. A. (2015). Correlational effect size benchmarks. Journal of Applied Psychology, 100, 431–449. 10.1037/a0038047 25314367

[bibr22-08902070251383955] Brader T. (2005). Striking a responsive chord: How political ads motivate and persuade voters by appealing to emotions. American Journal of Political Science, 49(2), 388–405. 10.1111/j.0092-5853.2005.00130.x

[bibr23-08902070251383955] (1) (2) (3) Brandt N. D. Savage C. Roberts B. W. Baumert J. Wagner J. (2022). Who do you trust? The role of level and change in trust and personality across young to middle adulthood for political interest and voting intentions. Journal of Research in Personality, 101(1), Article 104288. 10.1016/j.jrp.2022.104288

[bibr24-08902070251383955] Bromme L. Rothmund T. Azevedo F. (2022). Mapping political trust and involvement in the personality space—A meta‐analysis and new evidence. Journal of Personality, 90(6), 846–872. 10.1111/jopy.12700 35000199

[bibr25-08902070251383955] Burke D. L. Ensor J. Riley R. D. (2017). Meta‐analysis using individual participant data: One‐stage and two‐stage approaches, and why they may differ. Statistics in Medicine, 36(5), 855–875. 10.1002/sim.7141 27747915 PMC5297998

[bibr26-08902070251383955] Burkell J. Regan P. M. (2019). Voter preferences, voter manipulation, voter analytics: Policy options for less surveillance and more autonomy. Internet Policy Review, 8(4), 1–24. 10.14763/2019.4.1438

[bibr182-08902070251383955] Cafri G. Kromrey J. D. Brannick M. T. (2010). A meta-meta-analysis: Empirical review of statistical power, type I error rates, effect sizes, and model selection of meta-analyses published in psychology. Multivariate Behavioral Research, 45(2), 239–270. 10.1080/00273171003680187 26760285

[bibr27-08902070251383955] Caprara G. V. Schwartz S. Capanna C. Vecchione M. Barbaranelli C. (2006). Personality and politics: Values, traits, and political choice. Political Psychology, 27(1), 1–28. 10.1111/j.1467-9221.2006.00447.x

[bibr28-08902070251383955] Caprara G. V. Zimbardo P. G. (2004). Personalizing politics: A congruency model of political preference. American Psychologist, 59(7), 581–594. 10.1037/0003-066X.59.7.581 15491254

[bibr29-08902070251383955] Charles S. T. Gatz M. Kato K. Pedersen N. L. (2008). Physical health 25 years later: The predictive ability of neuroticism. Health Psychology: Official Journal of the Division of Health Psychology, American Psychological Association, 27(3), 369–378. 10.1037/0278-6133.27.3.369 18624602

[bibr30-08902070251383955] Cheung M. W.-L. (2014). Modeling dependent effect sizes with three-level meta-analyses: A structural equation modeling approach. Psychological Methods, 19(2), 211–229. 10.1037/a0032968 23834422

[bibr31-08902070251383955] Cohen P. Cohen J. Aiken L. S. West S. G. (1999). The problem of units and the circumstance for POMP. Multivariate Behavioral Research, 34(3), 315–346. 10.1207/S15327906MBR3403_2

[bibr32-08902070251383955] Connelly B. S. Ones D. S. Chernyshenko O. S. (2014). Introducing the special section on openness to experience: Review of openness taxonomies, measurement, and nomological net. Journal of Personality Assessment, 96(1), 1–16. 10.1080/00223891.2013.830620 24073877

[bibr33-08902070251383955] Conway J. M. Lance C. E. (2010). What reviewers should expect from authors regarding common method bias in organizational research. Journal of Business and Psychology, 25(3), 325–334. 10.1007/s10869-010-9181-6

[bibr34-08902070251383955] (1) (2) Cooper C. A. Golden L. Socha A. (2013). The Big Five personality factors and mass politics. Journal of Applied Social Psychology, 43(1), 68–82. 10.1111/j.1559-1816.2012.00982.x

[bibr35-08902070251383955] Coppedge M. Gerring J. Knutsen C. H. Lindberg S. I. Teorell J. Altman D. Bernhard M. Cornell A. Fish M. S. Gastaldi L. Gjerløw H. Glynn A. Grahn S. Hicken A. Kinzelbach K. Marquardt K. L. McMann K. Mechkova V. Neundorf A. Ziblatt D. (2023). V-Dem Dataset v13 [Data set]. Varieties of democracy (V-Dem) project. https://www.v-dem.net/data/the-v-dem-dataset/

[bibr36-08902070251383955] Costa P. T. Jr. McCrae R. R. (1976). Age differences in personality structure: A cluster analytic approach. Journal of Gerontology, 31(5), 564–570. 10.1093/geronj/31.5.564 950450

[bibr37-08902070251383955] Costa P. T. Jr. McCrae R. R. (1992). The NEO personality inventory-revised. Psychological Assessment Resources.

[bibr38-08902070251383955] Credé M. Harms P. Niehorster S. Gaye-Valentine A. (2012). An evaluation of the consequences of using short measures of the Big Five personality traits. Journal of Personality and Social Psychology, 102(4), 874–888. 10.1037/a0027403 22352328

[bibr39-08902070251383955] Crowe M. L. Lynam D. R. Miller J. D. (2018). Uncovering the structure of agreeableness from self-report measures. Journal of Personality, 86(5), 771–787. 10.1111/jopy.12358 29072788

[bibr40-08902070251383955] Cuijpers P. Smit F. Penninx B. W. de Graaf R. ten Have M. Beekman A. T. (2010). Economic costs of neuroticism: A population-based study. Archives of General Psychiatry, 67(10), 1086–1093. 10.1001/archgenpsychiatry.2010.130 20921124

[bibr41-08902070251383955] Curran P. J. Hussong A. M. (2009). Integrative data analysis: The simultaneous analysis of multiple data sets. Psychological Methods, 14(2), 81–100. 10.1037/a0015914 19485623 PMC2777640

[bibr42-08902070251383955] DeCoster J. Iselin A.-M. R. Gallucci M. (2009). A conceptual and empirical examination of justifications for dichotomization. Psychological Methods, 14(4), 349–366. 10.1037/a0016956 19968397

[bibr43-08902070251383955] Denny K. Doyle O. (2008). Political interest, cognitive ability and personality: Determinants of voter turnout in Britain. British Journal of Political Science, 38(2), 291–310. 10.1017/S000712340800015X

[bibr44-08902070251383955] DeYoung C. G. (2015). Openness/intellect: A dimension of personality reflecting cognitive exploration. In Mikulincer M. Shaver P. R. Cooper M. L. Larsen R. J. (Eds.), APA handbook of personality and social psychology, Vol. 4. Personality processes and individual differences (pp. 369–399). American Psychological Association. 10.1037/14343-017

[bibr45-08902070251383955] Egger M. Smith G. D. Schneider M. Minder C. (1997). Bias in meta-analysis detected by a simple, graphical test. BMJ, 315(7109), 629–634. 10.1136/bmj.315.7109.629 9310563 PMC2127453

[bibr46-08902070251383955] Erikson E. H. (1959). Identity and the life cycle, psychological issues monograph 1. International University Press.

[bibr47-08902070251383955] Fedorov V. Mannino F. Zhang R. (2009). Consequences of dichotomization. Pharmaceutical Statistics, 8(1), 50–61. 10.1002/pst.331 18389492

[bibr48-08902070251383955] Fisher R. A. (1921). On the ‘probable error’ of a coefficient of correlation deduced from a small sample. Metron, 1, 1–32.

[bibr49-08902070251383955] Flanagan C. Levine P. (2010). Civic engagement and the transition to adulthood. The Future of Children, 20(1), 159–179. 10.1353/foc.0.0043 20364626

[bibr50-08902070251383955] Freund A. M. Baltes P. B. (2002). Life-management strategies of selection, optimization and compensation: Measurement by self-report and construct validity. Journal of Personality and Social Psychology, 82(4), 642–662. 10.1037/0022-3514.82.4.642 11999929

[bibr51-08902070251383955] Freund A. M. Blanchard-Fields F. (2014). Age-related differences in altruism across adulthood: Making personal financial gain versus contributing to the public good. Developmental Psychology, 50(4), 1125–1136. 10.1037/a0034491 24059256

[bibr52-08902070251383955] Funder D. C. Ozer D. J. (2019). Evaluating effect size in psychological research: Sense and nonsense. Advances in Methods and Practices in Psychological Science, 2(2), 156–168. 10.1177/2515245919847202

[bibr53-08902070251383955] (1) (2) (3) Furnham A. Cheng H. (2019). Personality traits and socio-demographic variables as predictors of political interest and voting behavior in a British cohort. Journal of Individual Differences, 40(2), 118–125. 10.1027/1614-0001/a000283

[bibr54-08902070251383955] (1) (3) Gallego A. Oberski D. (2012). Personality and political participation: The mediation hypothesis. Political Behavior, 34(3), 425–451. 10.1007/s11109-011-9168-7

[bibr55-08902070251383955] Gelman A. Hill J. Yajima M. (2012). Why we (usually) don't have to worry about multiple comparisons. Journal of Research on Educational Effectiveness, 5(2), 189–211. 10.1080/19345747.2011.618213

[bibr56-08902070251383955] (1) (2) (3) Gerber A. S. Huber G. A. Doherty D. Dowling C. M. (2011). The Big Five personality traits in the political arena. Annual Review of Political Science, 14(1), 265–287. 10.1146/annurev-polisci-051010-111659

[bibr57-08902070251383955] (1) (2) (3) Gerber A. S. Huber G. A. Doherty D. Dowling C. M. Panagopoulos C. (2013). Big five personality traits and responses to persuasive appeals: Results from voter turnout experiments. Political Behavior, 35(4), 687–728. 10.1007/s11109-012-9216-y

[bibr58-08902070251383955] Gibney E. (2018, March 29). The scant science behind Cambridge Analytica’s controversial marketing techniques. Nature. 10.1038/d41586-018-03880-4

[bibr59-08902070251383955] Gignac G. Szodorai E. (2016). Effect size guidelines for individual differences researchers. Personality and Individual Differences, 102, 74–78. 10.1016/J.PAID.2016.06.069

[bibr60-08902070251383955] Gosling S. D. Rentfrow P. J. Swann W. B. Jr. (2003). A very brief measure of the Big-Five personality domains. Journal of Research in Personality, 37(6), 504–528. 10.1016/S0092-6566(03)00046-1

[bibr61-08902070251383955] Götz F. M. Gosling S. D. Rentfrow P. J. (2022). Small effects: The indispensable foundation for a cumulative psychological science. Perspectives on Psychological Science: A Journal of the Association for Psychological Science, 17(1), 205–215. 10.1177/1745691620984483 34213378

[bibr62-08902070251383955] Grassegger H. Krogerus M. (2017, January 28). The data that turned the world upside down. Motherboard. https://www.vice.com/en/article/how-our-likes-helped-trump-win/

[bibr63-08902070251383955] Graziano W. G. Eisenberg N. (1997). Agreeableness: A dimension of personality. In Hogan R. Johnson J. A. Briggs S. R. (Eds.), Handbook of personality psychology (pp. 795–824). Academic Press.

[bibr64-08902070251383955] Graziano W. G. Tobin R. M. (2009). Agreeableness. In Leary M. R. Hoyle R. H. (Eds.), Handbook of individual differences in social behavior (pp. 46–61). The Guilford Press.

[bibr65-08902070251383955] Habashi M. M. Graziano W. G. Hoover A. E. (2016). Searching for the prosocial personality: A Big Five approach to linking personality and prosocial behavior. Personality and Social Psychology Bulletin, 42(9), 1177–1192. 10.1177/0146167216652859 27401167

[bibr66-08902070251383955] Haehner P. Krämer M. D. Bleidorn W. Hopwood C. J. (2025). Validating the Big Five aspect scales (BFAS) and the short form (BFAS-S) in German, French, and Italian. 10.31234/osf.io/4p2mw_v1

[bibr67-08902070251383955] Haehner P. Wright A. J. Andrae R. Lubczyk T. Rosero Betancourt L. A. Hopwood C. J. Bleidorn W. (2025). A smartphone-based intervention to decrease neuroticism: Protocol of the CHILL Study. Personality Science, 6(1), Article 27000710251319409. 10.1177/27000710251319409

[bibr68-08902070251383955] Haehner P. Wright A. J. Bleidorn W. (2024). A systematic review of volitional personality change research. Communications Psychology, 2(1), 115. 10.1038/s44271-024-00167-5 39616261 PMC11608366

[bibr69-08902070251383955] Harder J. Krosnick J. A. (2008). Why do people vote? A psychological analysis of the causes of voter turnout. Journal of Social Issues, 64(3), 525–549. 10.1111/j.1540-4560.2008.00576.x

[bibr70-08902070251383955] Hedges L. V. Tipton E. (2010). Meta-analysis. In Steptoe A. (Ed.), Handbook of behavioral medicine: Methods and applications (pp. 909–921). Springer.

[bibr71-08902070251383955] Heene M. Bollmann S. Bühner M. (2014). Much ado about nothing, or much to do about something? Journal of Individual Differences, 35(4), 245–249. 10.1027/1614-0001/a000146

[bibr72-08902070251383955] Hemphill J. F. (2003). Interpreting the magnitudes of correlation coefficients. American Psychologist, 58(1), 78–79. 10.1037/0003-066X.58.1.78 12674822

[bibr73-08902070251383955] Henrich J. Heine S. J. Norenzayan A. (2010). The weirdest people in the world? Behavioral and Brain Sciences, 33(2/3), 61–135. 10.1017/S0140525X0999152X 20550733

[bibr74-08902070251383955] Henson R. K. (2001). Understanding internal consistency reliability estimates: A conceptual primer on coefficient alpha. Measurement and Evaluation in Counseling and Development, 34(3), 177–189. 10.1080/07481756.2002.12069034

[bibr75-08902070251383955] Hisler G. C. Krizan Z. DeHart T. Wright A. G. (2020). Neuroticism as the intensity, reactivity, and variability in day-to-day affect. Journal of Research in Personality, 87, Article 103964. 10.1016/j.jrp.2020.103964

[bibr184-08902070251383955] Hohn R. E. Slaney K. L. Tafreshi D. (2019). Primary study quality in psychological meta-analyses: An empirical assessment of recent practice. Frontiers in Psychology, 9, 2667. 10.3389/fpsyg.2018.02667 30687152 PMC6333691

[bibr76-08902070251383955] Holden R. R. (2010). Social desirability. In Corsini R. J. Craighead W. E. (Eds.), Encyclopedia of psychology. Wiley, pp 1628–1629.

[bibr77-08902070251383955] Hopwood C. J. Lenhausen M. R. Stahlmann A. G. Bleidorn W. (2022). Personality aspects and proenvironmental attitudes. Journal of Personality, 92(3), 784–799. 10.1111/jopy.12795 36401807

[bibr78-08902070251383955] Howard G. S. Maxwell S. E. Wiener R. L. Boynton K. S. Rooney W. M. (1980). Is a behavioral measure the best estimate of behavioral parameters? Perhaps not. Applied Psychological Measurement, 4(3), 293–311. 10.1177/014662168000400302

[bibr79-08902070251383955] (1) (2) (3) Huber B. Goyanes M. Gil de Zúñiga H. (2021). Linking extraversion to collective and individual forms of political participation: The mediating role of political discussion. Social Science Quarterly, 102(4), 1289–1310. 10.1111/ssqu.12978

[bibr80-08902070251383955] Hudson N. W. Fraley R. C. (2015). Volitional personality trait change: Can people choose to change their personality traits? Journal of Personality and Social Psychology, 109(3), 490–507. 10.1037/pspp0000021 25822032

[bibr81-08902070251383955] Hudson N. W. Roberts B. W. (2014). Goals to change personality traits: Concurrent links between personality traits, daily behavior, and goals to change oneself. Journal of Research in Personality, 53, 68–83. 10.1016/j.jrp.2014.08.008

[bibr82-08902070251383955] Hunter J. E. Schmidt F. L. (1982). Meta-analysis. In Hambleton R. K. Zaal J. N. (Eds.), Advances in educational and psychological testing: Theory and applications (pp. 157–183). Springer.

[bibr83-08902070251383955] (1) Ivanchenko A. Ignatieva I. Lefterov V. Timchenko O. (2019). Personality traits as determinants of political behavior: Ukrainian electoral and voting tendencies. Studia Politica. Romanian Political Science Review, 3–4, 127–151.

[bibr84-08902070251383955] John O. P. Donahue E. M. Kentle R. L. (1991). The Big Five inventory—Versions 4a and 54. University of California, Berkeley, Institute of Personality and Social Research.

[bibr85-08902070251383955] John O. P. Naumann L. P. Soto C. J. (2008). Paradigm shift to the integrative Big Five trait taxonomy: History, measurement, and conceptual issues. In John O. P. Robins R. W. Pervin L. A. (Eds.), Handbook of personality: Theory and research (pp. 114–158). The Guilford Press.

[bibr86-08902070251383955] Jokela M. Pulkki-Råback L. Elovainio M. Kivimäki M. (2014). Personality traits as risk factors for stroke and coronary heart disease mortality: Pooled analysis of three cohort studies. Journal of Behavioral Medicine, 37(5), 881–889. 10.1007/s10865-013-9548-z 24203126

[bibr87-08902070251383955] Kim H. Di Domenico S. I. Connelly B. S. (2019). Self–other agreement in personality reports: A meta-analytic comparison of self-and informant-report means. Psychological Science, 30(1), 129–138. 10.1177/0956797618810000 30481113

[bibr183-08902070251383955] Knapp G. Hartung J. (2003). Improved tests for a random effects meta-regression with a single covariate. Statistics in Medicine, 22, 2693–2710. 10.1002/sim.1482 12939780

[bibr88-08902070251383955] Konstabel K. Aavik T. Allik J. (2006). Social desirability and consensual validity of personality traits. European Journal of Personality, 20(7), 549–566. 10.1002/per.593

[bibr89-08902070251383955] Kosinski M. Matz S. C. Gosling S. D. Popov V. Stillwell D. (2015). Facebook as a research tool for the social sciences: Opportunities, challenges, ethical considerations, and practical guidelines. American Psychologist, 70(6), 543–556. 10.1037/a0039210 26348336

[bibr90-08902070251383955] Kotov R. Gamez W. Schmidt F. Watson D. (2010). Linking “big” personality traits to anxiety, depressive, and substance use disorders: A meta-analysis. Psychological Bulletin, 136(5), 768–821. 10.1037/a0020327 20804236

[bibr91-08902070251383955] Kretzschmar A. Gignac G. E. (2019). At what sample size do latent variable correlations stabilize? Journal of Research in Personality, 80, 17–22. 10.1016/j.jrp.2019.03.007

[bibr92-08902070251383955] Krotzek L. J. (2019). Inside the voter’s mind: The effect of psychometric microtargeting on feelings toward and propensity to vote for a candidate. International Journal of Communication, 13, 3609–3629.

[bibr93-08902070251383955] Lahey B. B. (2009). Public health significance of neuroticism. American Psychologist, 64(4), 241–256. 10.1037/a0015309 19449983 PMC2792076

[bibr94-08902070251383955] Larson R. B. (2019). Controlling social desirability bias. International Journal of Market Research, 61(5), 534–547. 10.1177/1470785318805305

[bibr95-08902070251383955] (1) Lynch B. N. (2008). The associations among personality factors, the theory of planned behavior and voting. [Master’s thesis]. California State University. Sac State Scholars. https://hdl.handle.net/10211.9/1284

[bibr96-08902070251383955] MacCallum R. C. Zhang S. Preacher K. J. Rucker D. D. (2002). On the practice of dichotomization of quantitative variables. Psychological Methods, 7(1), 19–40. 10.1037/1082-989X.7.1.19 11928888

[bibr97-08902070251383955] Magnus K. Diener E. Fujita F. Pavot W. (1993). Extraversion and neuroticism as predictors of objective life events: A longitudinal analysis. Journal of Personality and Social Psychology, 65(5), 1046–1053. 10.1037/0022-3514.65.5.1046 8246112

[bibr98-08902070251383955] Malouff J. M. Thorsteinsson E. B. Rooke S. E. Schutte N. S. (2007). Alcohol involvement and the five-factor model of personality: A meta-analysis. Journal of Drug Education, 37(3), 277–294. 10.2190/DE.37.3.d 18047183

[bibr99-08902070251383955] Malouff J. M. Thorsteinsson E. B. Schutte N. S. Bhullar N. Rooke S. E. (2010). The Five-Factor model of personality and relationship satisfaction of intimate partners: A meta-analysis. Journal of Research in Personality, 44(1), 124–127. 10.1016/j.jrp.2009.09.004

[bibr100-08902070251383955] Manzetti L. Wilson C. J. (2007). Why do corrupt governments maintain public support? Comparative Political Studies, 40(8), 949–970. 10.1177/0010414005285759

[bibr101-08902070251383955] Marcus G. E. MacKuen M. B. (1993). Anxiety, enthusiasm, and the vote: The emotional underpinnings of learning and involvement during presidential campaigns. American Political Science Review, 87(3), 672–685. 10.2307/2938743

[bibr102-08902070251383955] (1) (2) (3) Mattila M. Wass H. Söderlund P. Fredriksson S. Fadjukoff P. Kokko K. (2011). Personality and turnout: Results from the Finnish longitudinal studies. Scandinavian Political Studies, 34(4), 287–306. 10.1111/j.1467-9477.2011.00273.x

[bibr103-08902070251383955] Matz S. C. Beck E. D. Atherton O. E. White M. Rauthmann J. F. Mroczek D. K. Kim M. Bogg T. (2023). Personality science in the digital age: The promises and challenges of psychological targeting for personalized behavior-change interventions at scale. Perspectives on Psychological Science: A Journal of the Association for Psychological Science, 19(6), 1031–1056. 10.1177/17456916231191774 37642145

[bibr104-08902070251383955] Matz S. C. Kosinski M. Nave G. Stillwell D. J. (2017). Psychological targeting as an effective approach to digital mass persuasion. Proceedings of the National Academy of Sciences of the United States of America, 114(48), 12714–12719. 10.1073/pnas.1710966114 29133409 PMC5715760

[bibr105-08902070251383955] Matz S. C. Netzer O. (2017). Using big data as a window into consumers’ psychology. Current Opinion in Behavioral Sciences, 18, 7–12. 10.1016/j.cobeha.2017.05.009

[bibr106-08902070251383955] McAdams D. P. (2001). Generativity in midlife. In Lachman M. E. (Ed.), Handbook of midlife development (pp. 395–446). Wiley.

[bibr107-08902070251383955] McCrae R. R. Costa P. T. Jr. Ostendorf F. Angleitner A. Hřebíčková M. Avia M. D. Sanz J. Sánchez-Bernardos M. L. Kusdil M. E. Woodfield R. Saunders P. R. Smith P. B. (2000). Nature over nurture: Temperament, personality, and life span development. Journal of Personality and Social Psychology, 78(1), 173–186. 10.1037/0022-3514.78.1.173 10653513

[bibr108-08902070251383955] McShane B. B. Gal D. Gelman A. Robert C. Tackett J. L. (2019). Abandon statistical significance. The American Statistician, 73(sup1), 235–245. 10.1080/00031305.2018.1527253

[bibr109-08902070251383955] Metzger A. Alvis L. M. Oosterhoff B. Babskie E. Syvertsen A. Wray-Lake L. (2018). The intersection of emotional and sociocognitive competencies with civic engagement in middle childhood and adolescence. Journal of Youth and Adolescence, 47(8), 1663–1683. 10.1007/s10964-018-0842-5 29572778

[bibr110-08902070251383955] Miller J. D. Gaughan E. T. Maples J. Price J. (2011). A comparison of agreeableness scores from the Big Five Inventory and the NEO PI-R: Consequences for the study of narcissism and psychopathy. Assessment, 18(3), 335–339. 10.1177/1073191111411671 21665883

[bibr111-08902070251383955] (1) (2) Mondak J. Halperin K. (2008). A framework for the study of personality and political behaviour. British Journal of Political Science, 38(2), 335–362. 10.1017/S0007123408000173

[bibr112-08902070251383955] (1) (2) (3) Mondak J. J. Hibbing M. V. Canache D. Seligson M. A. Anderson M. R. (2010). Personality and civic engagement: An integrative framework for the study of trait effects on political behavior. American Political Science Review, 104(1), 85–110. 10.1017/S0003055409990359

[bibr113-08902070251383955] Mõttus R. (2016). Towards more rigorous personality trait–outcome research. European Journal of Personality, 30(4), 292–303. 10.1002/per.2041

[bibr114-08902070251383955] Mõttus R. Kandler C. Bleidorn W. Riemann R. McCrae R. R. (2017). Personality traits below facets: The consensual validity, longitudinal stability, heritability, and utility of personality nuances. Journal of Personality and Social Psychology, 112(3), 474–490. 10.1037/pspp0000100 27124378

[bibr115-08902070251383955] Nederhof A. J. (1985). Methods of coping with social desirability bias: A review. European Journal of Social Psychology, 15(3), 263–280. 10.1002/ejsp.2420150303

[bibr116-08902070251383955] Noftle E. E. Robins R. W. (2007). Personality predictors of academic outcomes: Big five correlates of GPA and SAT scores. Journal of Personality and Social Psychology, 93(1), 116–130. 10.1037/0022-3514.93.1.116 17605593

[bibr117-08902070251383955] Nosek B. A. Hardwicke T. E. Moshontz H. Allard A. Corker K. S. Dreber A. Fidler F. Hilgard J. Kline Struhl M. Nuijten M. B. Rohrer J. M. Romero F. Scheel A. M. Scherer L. D. Schönbrodt F. D. Vazire S. (2022). Replicability, robustness, and reproducibility in psychological science. Annual Review of Psychology, 73(1), 719–748. 10.1146/annurev-psych-020821-114157 34665669

[bibr118-08902070251383955] Nunnally J. C. Bernstein I. H. (1994). Psychometric theory. McGraw-Hill.

[bibr119-08902070251383955] Nyklíček I. Irrmischer M. (2017). For whom does mindfulness-based stress reduction work? Moderating effects of personality. Mindfulness, 8(4), 1106–1116. 10.1007/s12671-017-0687-0 28757903 PMC5506177

[bibr120-08902070251383955] Olaru G. Stieger M. Rüegger D. Kowatsch T. Flückiger C. Roberts B. W. Allemand M. (2022). Personality change through a digital-coaching intervention: Using measurement invariance testing to distinguish between trait domain, facet, and nuance change. European Journal of Personality, 8(2), 141–157. 10.1177/08902070221145088 PMC13021088 41908316

[bibr185-08902070251383955] Omoto A. M. Packard C. D. Ballew M. T. (2020). Personality and Volunteerism. In Carducci B. J. Nave C. S. Mio J. S. Mio J. S. Riggio R. E. (Eds.), The Wiley encyclopedia of personality and individual differences (pp. 477–451). John Wiley & Sons Ltd. 10.1002/9781119547181.ch339

[bibr121-08902070251383955] Ones D. S. Viswesvaran C. Reiss A. D. (1996). Role of social desirability in personality testing for personnel selection: The red herring. Journal of Applied Psychology, 81(6), 660–679. 10.1037//0021-9010.81.6.660

[bibr122-08902070251383955] Ozer D. J. Benet-Martínez V. (2006). Personality and the prediction of consequential outcomes. Annual Review of Psychology, 57, 401–421. 10.1146/annurev.psych.57.102904.190127 16318601

[bibr123-08902070251383955] Page M. J. McKenzie J. E. Bossuyt P. M. Boutron I. Hoffmann T. C. Mulrow C. D. Shamseer L. Tetzlaff J. M. Akl E. A. Brennan S. E. Chou R. Glanville J. Grimshaw J. M. Hróbjartsson A. Lalu M. M. Li T. Loder E. W. Mayo-Wilson E. McDonald S. Moher D. (2021). The PRISMA 2020 statement: An updated guideline for reporting systematic reviews. International Journal of Surgery, 88, Article 105906. 10.1016/j.ijsu.2021.105906 33789826

[bibr124-08902070251383955] Pastor D. A. Lazowski R. A. (2018). On the multilevel nature of meta-analysis: A tutorial, comparison of software programs, and discussion of analytic choices. Multivariate Behavioral Research, 53(1), 74–89. 10.1080/00273171.2017.1365684 28952787

[bibr125-08902070251383955] Perneger T. V. (1998). What’s wrong with Bonferroni adjustments. BMJ, 316(7139), 1236–1238. 10.1136/bmj.316.7139.1236 9553006 PMC1112991

[bibr126-08902070251383955] Peters J. L. Sutton A. J. Jones D. R. Abrams K. R. Rushton L. (2008). Contour-enhanced meta-analysis funnel plots help distinguish publication bias from other causes of asymmetry. Journal of Clinical Epidemiology, 61(10), 991–996. 10.1016/j.jclinepi.2007.11.010 18538991

[bibr127-08902070251383955] Podsakoff P. M. MacKenzie S. B. Lee J.-Y. Podsakoff N. P. (2003). Common method biases in behavioral research: A critical review of the literature and recommended remedies. Journal of Applied Psychology, 88(5), 879–903. 10.1037/0021-9010.88.5.879 14516251

[bibr128-08902070251383955] Poropat A. E. (2009). A meta-analysis of the five-factor model of personality and academic performance. Psychological Bulletin, 135(2), 322–338. 10.1037/a0014996 19254083

[bibr129-08902070251383955] (1) Pruysers S. Blais J. Chen P. G. (2019). Who makes a good citizen? The role of personality. Personality and Individual Differences, 146(5), 99–104. 10.1016/j.paid.2019.04.007

[bibr130-08902070251383955] Pustejovsky J. (2023). clubSandwich: Cluster-robust (sandwich) variance estimators with small-sample corrections (R package version 0.5.10). https://cran.r-project.org/package=clubSandwich

[bibr131-08902070251383955] R Core Team . (2023). R: A language and environment for statistical computing (Version 4.3.0). https://www.R-project.org/

[bibr132-08902070251383955] Revelle W. Condon D. M. (2019). Reliability from α to ω: A tutorial. Psychological Assessment, 31(12), 1395–1411. 10.1037/pas0000754 31380696

[bibr133-08902070251383955] Roberts B. W. Lejuez C. Krueger R. F. Richards J. M. Hill P. L. (2014). What is conscientiousness and how can it be assessed? Developmental Psychology, 50(5), 1315–1330. 10.1037/a0031109 23276130

[bibr134-08902070251383955] Roberts B. W. Luo J. Briley D. A. Chow P. I. Su R. Hill P. L. (2017). A systematic review of personality trait change through intervention. Psychological Bulletin, 143(2), 117–141. 10.1037/bul0000088 28054797

[bibr135-08902070251383955] Rothman K. J. (1990). No adjustments are needed for multiple comparisons. Epidemiology, 1(1), 43–46. 2081237

[bibr136-08902070251383955] Satterthwaite F. E. (1946). An approximate distribution of estimates of variance components. Biometrics, 2(6), 110–114. 10.2307/3002019 20287815

[bibr137-08902070251383955] Scherpenzeel A. C. Das M. (2011). “True” longitudinal and probability-based Internet panels: Evidence from The Netherlands. In Das M. Ester P. Kaczmirek L. (Eds.), Social and behavioral research and the Internet: Advances in applied methods and research strategies (pp. 77–104). Routledge/Taylor & Francis Group.

[bibr138-08902070251383955] Schmidt F. L. Hunter J. E. (2014). Methods of meta-analysis: Correcting error and bias in research findings (3rd ed.). Sage publications.

[bibr139-08902070251383955] (1) (2) (3) Schoen H. Steinbrecher M. (2013). Beyond total effects: Exploring the interplay of personality and attitudes in affecting turnout in the 2009 German federal election. Political Psychology, 34(4), 533–552. 10.1111/pops.12168

[bibr140-08902070251383955] Schwaba T. Rhemtulla M. Hopwood C. J. Bleidorn W. (2020). A facet atlas: Visualizing networks that describe the blends, cores, and peripheries of personality structure. PLoS One, 15(7), Article e0236893. 10.1371/journal.pone.0236893 32730328 PMC7392538

[bibr186-08902070251383955] Sheeran P. Webb T. L. (2016). The intention-behavior gap. Social and Personality Psycholog Compass, 10(9), 503–518. 10.1111/spc3.12265

[bibr141-08902070251383955] Sibley C. G. Osborne D. Duckitt J. (2012). Personality and political orientation: Meta-analysis and test of a threat-constraint model. Journal of Research in Personality, 46(6), 664–677. 10.1016/j.jrp.2012.08.002

[bibr187-08902070251383955] Sidik K. Jonkman J. N. (2002). A simple confidence interval for meta-analysis. Statistics in Medicine, 21, 3153–3159. 10.1002/sim.1262 12375296

[bibr142-08902070251383955] Simonsohn U. Simmons J. P. Nelson L. D. (2020). Specification curve analysis. Nature Human Behaviour, 4(11), 1208–1214. 10.1038/s41562-020-0912-z 32719546

[bibr143-08902070251383955] (1) (2) (3) Sindermann C. Montag C. (2023). Individual differences in need satisfaction and intentions to vote for specific political parties–results from Germany. Current Psychology, 42(11), 9496–9508. 10.1007/s12144-021-02100-z

[bibr144-08902070251383955] (1) (2) (3) Sindermann C. Mõttus R. Rozgonjuk D. Montag C. (2021). Predicting current voting intentions by big five personality domains, facets, and nuances–a random forest analysis approach in a German sample. Personality Science, 2, Article e6017. 10.5964/ps.6017

[bibr145-08902070251383955] Sleep C. E. Lynam D. R. Miller J. D. (2021). A comparison of the validity of very brief measures of the Big Five/Five-Factor Model of personality. Assessment, 28(3), 739–758. 10.1177/1073191120939160 32762351

[bibr146-08902070251383955] Smillie L. D. Kern M. L. Uljarevic M. (2019). Extraversion: Description, development, and mechanisms. In McAdams D. P. Shiner R. L. Tackett J. L. (Eds.), Handbook of personality development (pp. 118–136). The Guilford Press.

[bibr147-08902070251383955] Smith G. T. McCarthy D. M. Anderson K. G. (2000). On the sins of short-form development. Psychological Assessment, 12(1), 102–111. 10.1037/1040-3590.12.1.102 10752369

[bibr148-08902070251383955] (1) (2) (3) Smith K. B. Hibbing M. V. Hibbing J. R. (2019). Friends, relatives, sanity, and health: The costs of politics. PLoS One, 14(9), Article e0221870. 10.1371/journal.pone.0221870 31553726 PMC6760758

[bibr149-08902070251383955] Soto C. J. John O. P. (2019). Optimizing the length, width, and balance of a personality scale: How do internal characteristics affect external validity? Psychological Assessment, 31(4), 444–459. 10.1037/pas0000586 29792501

[bibr150-08902070251383955] Soutter A. R. B. Bates T. C. Mõttus R. (2020). Big Five and HEXACO personality traits, proenvironmental attitudes, and behaviors: A meta-analysis. Perspectives on Psychological Science: A Journal of the Association for Psychological Science, 15(4), 913–941. 10.1177/1745691620903019 32384257 PMC7333518

[bibr151-08902070251383955] Stahlmann A. G. Hopwood C. J. Bleidorn W. (2023). Big Five personality traits predict small but robust differences in civic engagement. Journal of Personality, 92(2), 480–494. 10.1111/jopy.12838 37066516

[bibr152-08902070251383955] Stanley T. D. Doucouliagos H. (2014). Meta‐regression approximations to reduce publication selection bias. Research Synthesis Methods, 5(1), 60–78. 10.1002/jrsm.1095 26054026

[bibr153-08902070251383955] Stephanopoulos N. O. McGhee E. M. (2015). Partisan gerrymandering and the efficiency gap. University of Chicago Law Review, 82(2), 831–900. https://www.jstor.org/stable/43410706

[bibr154-08902070251383955] Stieger M. Nißen M. Rüegger D. Kowatsch T. Flückiger C. Allemand M. (2018). PEACH, a smartphone-and conversational agent-based coaching intervention for intentional personality change: Study protocol of a randomized, wait-list controlled trial. BMC Psychology, 6(1), 43. 10.1186/s40359-018-0257-9 30180880 PMC6123904

[bibr155-08902070251383955] Stockemer D. (2017). What affects voter turnout? A review article/meta-analysis of aggregate research. Government and Opposition, 52(4), 698–722. 10.1017/gov.2016.30

[bibr156-08902070251383955] Strickhouser J. E. Zell E. Krizan Z. (2017). Does personality predict health and well-being? A metasynthesis. Health Psychology: Official Journal of the Division of Health Psychology, American Psychological Association, 36(8), 797–810. 10.1037/hea0000475 28277701

[bibr188-08902070251383955] Taylor S. Todd P. (1995). Decomposition and crossover effects in the theory of planned behavior: A study of consumer adoption intentions. International Journal of Research in Marketing, 12(2), 137–155. 10.1016/0167-8116(94)00019-K

[bibr157-08902070251383955] Terracciano A. Costa P. T. Jr. (2004). Smoking and the Five‐Factor model of personality. Addiction, 99(4), 472–481. 10.1111/j.1360-0443.2004.00687.x 15049747 PMC2376761

[bibr158-08902070251383955] Thalmayer A. G. Saucier G. Eigenhuis A. (2011). Comparative validity of brief to medium-length Big Five and Big Six personality questionnaires. Psychological Assessment, 23(4), 995–1009. 10.1037/a0024165 21859221

[bibr159-08902070251383955] Thalmayer A. G. Toscanelli C. Arnett J. J. (2021). The neglected 95% revisited: Is American psychology becoming less American? American Psychologist, 76(1), 116–129. 10.1037/amp0000622 32271027

[bibr160-08902070251383955] Thielmann I. de Vries R. E. (2021). Who wants to change and how? On the trait-specificity of personality change goals. Journal of Personality and Social Psychology, 121(5), 1112–1139. 10.1037/pspp0000304 33475400

[bibr161-08902070251383955] Thielmann I. Spadaro G. Balliet D. (2020). Personality and prosocial behavior: A theoretical framework and meta-analysis. Psychological Bulletin, 146(1), 30–90. 10.1037/bul0000217 31841013

[bibr162-08902070251383955] Tipton E. Pustejovsky J. E. (2015). Small-sample adjustments for tests of moderators and model fit using robust variance estimation in meta-regression. Journal of Educational and Behavioral Statistics, 40(6), 604–634. 10.3102/1076998615606099

[bibr163-08902070251383955] (1) Turska-Kawa A. (2013). Big five personality traits model in electoral behaviour studies. Romanian Journal of Political Sciences, 13(02), 69–105.

[bibr164-08902070251383955] (1) (3) Verhulst B. (2012). Integrating classical and contemporary explanations of political participation. Twin Research and Human Genetics: The Official Journal of the International Society for Twin Studies, 15(1), 42–51. 10.1375/twin.15.1.42 22784452 PMC3756812

[bibr165-08902070251383955] Viechtbauer W. (2023). Metafor: Meta-analysis package for R (R package version 4.4-0). https://cran.r-project.org/package=metafor

[bibr166-08902070251383955] Viechtbauer W. Cheung M. W. L. (2010). Outlier and influence diagnostics for meta‐analysis. Research Synthesis Methods, 1(2), 112–125. 10.1002/jrsm.11 26061377

[bibr167-08902070251383955] Voracek M. Kossmeier M. Tran U. S. (2019). Which data to meta-analyze, and how? Zeitschrift für Psychologie, 227(1), 64–82. 10.1027/2151-2604/a000357

[bibr168-08902070251383955] Wang C. H. (2014). Gender differences in the effects of personality traits on voter turnout. Electoral Studies, 34, 167–176. 10.1016/j.electstud.2013.10.005

[bibr169-08902070251383955] Wang C. H. Weng D. L. C. Cha H. J. (2017). Personality traits and voter turnout in South Korea: The mediation argument. Japanese Journal of Political Science, 18(3), 426–445. 10.1017/S146810991700010X

[bibr170-08902070251383955] (1) (2) Wang C. H. Weng D. L. C. Tsai C. H. (2019). Personality traits and political participation in Taiwan: A mediation approach. Political Science, 71(3), 175–192. 10.1080/00323187.2020.1767506

[bibr171-08902070251383955] (1) (3) Weinschenk A. (2013). ’Cause you’ve got personality: Political participation and the tendency to join civic groups. Sage Open, 3(4), Article 2158244013508418. 10.1177/2158244013508418

[bibr172-08902070251383955] Weinschenk A. C. (2017). Big five personality traits, political participation, and civic engagement: Evidence from 24 countries. Social Science Quarterly, 98(5), 1406–1421. 10.1111/ssqu.12380

[bibr173-08902070251383955] Weinschenk A. C. Dawes C. T. (2018). Genes, personality traits, and the sense of civic duty. American Politics Research, 46(1), 47–76. 10.1177/1532673X17710760

[bibr174-08902070251383955] Weinschenk A. C. Panagopoulos C. Drabot K. van der Linden S. (2018). Gender and social conformity: Do men and women respond differently to social pressure to vote? Social Influence, 13(2), 53–64. 10.1080/15534510.2018.1432500

[bibr175-08902070251383955] (1) (3) Weinschenk A. C. Dawes C. T. Kandler C. Bell E. Riemann R. (2019). New evidence on the link between genes, psychological traits, and political engagement. Politics and the Life Sciences: The Journal of the Association for Politics and the Life Sciences, 38(1), 1–13. 10.1017/pls.2019.3 31094675

[bibr176-08902070251383955] (1) Weinschenk A. C. Panagopoulos C. (2014). Personality, negativity, and political participation. Journal of Social and Political Psychology, 2(1), 164–182. 10.5964/jspp.v2i1.280

[bibr177-08902070251383955] Widiger T. A. Oltmanns J. R. (2017). Neuroticism is a fundamental domain of personality with enormous public health implications. World Psychiatry: Official Journal of the World Psychiatric Association (WPA), 16(2), 144–145. 10.1002/wps.20411 28498583 PMC5428182

[bibr178-08902070251383955] Wilt J. Revelle W. (2017). Extraversion. In Widiger T. A. (Ed.), The Oxford handbook of the five factor model (pp. 57–81). Oxford University Press.

[bibr179-08902070251383955] Wright A. J. Haehner P. Hopwood C. J. Bleidorn W. (2024). A systematic review and taxonomy of neuroticism interventions for the general public. 10.31234/osf.io/jy3eb

[bibr180-08902070251383955] Zarouali B. Dobber T. De Pauw G. de Vreese C. (2022). Using a personality-profiling algorithm to investigate political microtargeting: Assessing the persuasion effects of personality-tailored ads on social media. Communication Research, 49(8), 1066–1091. 10.1177/0093650220961965

